# Human Umbilical Cord Mesenchymal Stem Cells Modulate Cytokine Secretion of CD4^+^ T Cell in Systemic Lupus Erythematosus by Inhibiting HSP90AA1 in the Glucose‐Activated PI3K‐AKT Pathway

**DOI:** 10.1002/iid3.70239

**Published:** 2025-08-13

**Authors:** Lu Jin, Meng Ding, Shaoxin Cui, Lin Yang, Jingjing He, Xiaoping Wang, Fei Chang, Jingjing Yu, Yiming Yang, Hongtao Jin, Min Shi, Jun Ma, Aijing Liu

**Affiliations:** ^1^ Department of Rheumatology and Immunology The Second Hospital of Hebei Medical University Shijiazhuang Hebei China; ^2^ Hebei International Joint Research Center on Rheumatic Diseases Shijiazhuang Hebei China; ^3^ Department of Respiratory and Critical Care Medicine The Second Hospital of Hebei Medical University Shijiazhuang Hebei China; ^4^ Hebei Medical University‐University of Galway Stem Cell Research Center, Hebei Medical University Shijiazhuang Hebei China; ^5^ Hebei Key Laboratory of Laboratory Medicine Shijiazhuang Hebei China; ^6^ Hebei Research Center for Stem Cell Medical Translational Engineering Shijiazhuang Hebei China

**Keywords:** CD4^+^ T cell, cytokine, glucose metabolism, HSP90AA1, Lupus, mesenchymal stem cell, PI3K‐AKT pathway

## Abstract

**Objective:**

Treatment with human umbilical cord mesenchymal stem cells (hUC‐MSCs) attenuated the clinical manifestations of systemic lupus erythematosus (SLE). We investigated the metabolic mechanism whereby hUC‐MSCs modify CD4^+^ T cell cytokine secretion in lupus.

**Methods:**

The study enrolled 30 untreated lupus patients and 20 sex, age, and body mass index matched healthy controls (HCs). CD4^+^ T cells were isolated by magnetic sorting, and stimulated with anti‐CD3/CD28. The hUC‐MSCs treatment (MSCT) groups were coculturing hUC‐MSCs to CD4^+^ T cells from moderate and severe SLE (SLE‐MS) groups for 72 h at ratios of 1:25 (T1), 1:10 (T2), and 1:5 (T3). Cytokine concentration and proliferation of the CD4^+^ T cells were measured by Luminex liquid chip assay and cell counting kit‐8, respectively. Glucose metabolic capacity was measured by Seahorse real‐time metabolic analysis. The role of hUC‐MSCs on cytokine secretion was analyzed by transcriptome sequencing. Glucose enzymes levels and HSP90AA1/PI3K/AKT pathway activity were analyzed by real‐time quantitative PCR and western blot. The CD4^+^ T cell subsets were detected by flow cytometry.

**Results:**

Compared with HCs, the enhanced glycolysis and mitochondrial oxygen consumption of SLE‐CD4^+^ T cells were positively associated with disease activity. Treatment with hUC‐MSCs proportionally decreased glucose metabolism and proliferation of SLE‐CD4^+^ T cells. The hUC‐MSCs treatment significantly diminished supernatant concentrations of interferon‐γ, tumor necrosis factor‐α, interleukin (IL)‐4, and IL‐17 in SLE‐MS group, as well as inhibited HSP90AA1 in the glucose‐activated PI3K‐AKT pathway. In animal experiment, the systemic administration of hUC‐MSCs and inhibition of HSP90AA1 resulted in a reduction of glucose metabolites, enzymes, pro‐inflammatory factor levels, and HSP90AA1*/*PI3K/AKT signaling pathway activity.

**Conclusions:**

The hUC‐MSCs treatment inhibited overactive glucose metabolism of SLE‐CD4^+^ T cells. HSP90AA1 in the PI3K‐AKT pathway induced by the glucose metabolism may be involved in the anti‐inflammatory function of hUC‐MSCs treatment.

## 1 Introduction

Systemic lupus erythematosus (SLE) is a systemic autoimmune disease involving a widespread tissue inflammation. T lymphocyte dysfunction and inflammatory factors are involved in the pathogenesis of SLE [1].

CD4^+^ T cells are a crucial component of the adaptive immune system, primarily responsible for the proliferation and differentiation of effector cells, as well as the secretion of cytokines. The major classical subsets of CD4^+^ T cells include T helper (Th)1, Th2, Th17, and regulatory T (Treg) cells. Studies have confirmed that patients with lupus nephritis or active SLE exhibit an increased proportion of Th1 and Th17 cells [2, 3]. As SLE enters remission, there is the percentage of Treg cells gradually increases [4]. Existing research has demonstrated a positive correlation between the Th17/Treg cell ratio and SLE activity [5, 6]. Th1 and Th2 cells are two extensively studied Th subsets playing essential regulator roles in inflammatory response associated with SLE. However, the role of the Th1/Th2 cell balance in the progression of SLE remains a subject of controversy [3, 6, 7]. Collectively, investigations focusing on CD4^+^ T cell subsets in SLE hold significant clinical application value.

Imbalances in the levels of pro‐and anti‐inflammatory cytokines secreted by various CD4^+^ T cell subsets play a critical role in the disruption of immune tolerance and the exacerbation of inflammation [8]. Th1 cytokines, which include interferon (IFN)‐γ, tumor necrosis factor (TNF)‐α, granulocyte‐macrophage colony‐stimulating factor (GM‐CSF), and interleukin (IL)‐2, are essential for inflammation and B cell activation [3, 9]. Th17 cytokines implicated in SLE include IL‐17 and IL‐23, both of which are strongly association with anti‐dsDNA antibody titers and immunoglobulin G (IgG) production [10, 11]. IL‐4 and IL‐10 are classical Th2 cytokines that exert immunomodulatory effects on immune response, B cell antigen presentation, and T cell proliferation [7, 12]. Transforming growth factor (TGF) β is an important anti‐inflammatory cytokine known for its immunosuppressive properties as well as its ability to restore immune tolerance, which produced by Tregs and Th2 cells [8]. Extensive research has demonstrated dysregulated cytokines levels correlated with alterations in SLE activity [2, 3, 8].

The glucose metabolic pathway of CD4^+^ T cell is correlated with cell activation status [13]. At homeostasis, quiescent CD4^+^ T cell primarily relies on mitochondrial oxidative phosphorylation to uphold cell morphology and basal bioenergetic requirements. Upon CD4^+^ T cell activation, T cell receptor binding and co‐stimulated signaling increase glucose uptake and upregulate glycolysis and oxidative phosphorylation [14]. In SLE, activated CD4^+^ T cells exhibit a glucose metabolic transition from mitochondrial oxidative phosphorylation to aerobic glycolysis to support their greater bioenergetic demands, such as cellular differentiation, proliferation and cytokine secretion [15, 16]. Current research on CD4^+^ T cell glucose metabolic reprogramming in SLE is focused primarily on cellular differentiation and proliferation. However, still unclear is the metabolic mechanism of forming a pro‐inflammatory milieu of lupus.

Because of the limitations and adverse events of general and targeted treatment of SLE, there is an urgent need for new strategies with improved efficacy and reduced toxicity. Mesenchymal stem cell (MSC) treatment is a new therapeutic option with unique advantages, including reduction of inflammation, immunomodulatory effects, and tissue repair [17, 18, 19]. Human umbilical cord‐derived MSCs (hUC‐MSCs) are multipotent stem cells, and can be differentiated into hepatocytes, osteocytes, or chondrocytes in certain culture medium [20]. The application of hUC‐MSCs had a clinical potential of SLE remission by regulating T lymphocyte proliferation, re‐establishing the balance of T lymphocyte subsets, and modulating inflammatory factor levels [21, 22, 23]. However, how hUC‐MSCs treatment alter the inflammatory microenvironment of SLE‐CD4^+^ T cell is poorly understood.

In this study, we focused our study on changes in glucose metabolism of CD4^+^ T cells and inflammatory factors expression following hUC‐MSCs treatment to identify the metabolic mechanism of hUC‐MSCs therapy on CD4^+^ T cell cytokine secretion in lupus.

## 2 Methods

### 2.1 Study Subjects

A total of 30 untreated patients with SLE were recruited for our study from the Department of Rheumatology and Immunology of the Second Hospital of Hebei Medical University between January 2023 to September 2023. SLE definition met the revised ACR and the SLICC criteria [24]. Patients were excluded if they had any of the following conditions: (1) Infectious, metabolic, or other autoimmune diseases, (2) Severe life‐threatening diseases (e.g., malignancy, organ failure), (3) Metformin use, (4) History of graft‐versus‐host disease or allergic diseases, (5) Prior radiotherapy for face, head, or neck. All participants signed written informed consent before obtaining peripheral blood samples. Details that might disclose the identity of participants were expunged. The study enrolled 20 age, sex, and body mass index (BMI) matched healthy volunteers as controls. This study adhered strictly to the World Medical Association Declaration of Helsinki.

Clinical and demographic characteristics were collected, including basic information (e.g., age, sex, height, and weight), systemic involvement (e.g., rash, arthritis, hematological system, and urinary system), and autoimmune parameters (e.g., anti‐dsDNA antibody, complements, and immunoglobulins). The Systemic Lupus Erythematosus Disease Activity Index‐2000 (SLEDAI‐2K) was used for clinical severity assessment, in which severe activity group (*n* **=** 7) was defined as SLEDAI‐2K > 12, moderate (*n* **=** 11) as 7 ≤ SLEDAI‐2K ≤ 12 and mild (*n* **=** 12) as 1 ≤ SLEDAI‐2 K ≤ 6.

### 2.2 Experimental Animals

To verify the role of HSP90AA1 in inhibiting the secretion of inflammatory factors of CD4^+^ T cells from SLE compared with healthy controls (HCs), we use a MRL/lpr lupus‐prone mouse model, and C57BL/6 mice served as the HC group. Eight‐week‐old female MRL/lpr mice and C57BL/6 mice were obtained from SiPeiFu Biotech Co. Ltd (Beijing, China). The mice were housed in a specific pathogen‐free environment at the Animal Experimental Center of Hebei Medical University, maintained at a room temperature of 25** ±** 2°C, relative humidity of 65** ±** 2%, and subjected to a 12 h light/dark cycle. Animal experiments were conducted in accordance with the National Institutes of Health Guide for Care and Use of Laboratory Animals and received approval from the Research Ethics Committee of the Second Hospital of Hebei Medical University (2023‐AE‐164).

### 2.3 Treatment of Mice

For in vivo animal experiments, a total of 10 female MRL/lpr mice were utilized as models for experimental SLE and were randomly divided into two groups at 8 weeks of age as follows, phosphate buffered saline (PBS) group (*n* **=** 5) and hUC‐MSCs group (*n* **=** 5). Five female C57BL/6 mice served as normal control models (*n* **=** 5). The MRL/lpr mice in hUC‐MSCs group received a tail vein injection (TVI) of hUC‐MSCs (8 × 10^5^ cells, 200 µL) on Weeks 16 and 18. PBS was used as vehicle control for both PBS group and C57BL/6 mice.

For in vitro animal experiments, an additional cohort consisting of five female MRL/lpr mice and five female C57BL/6 mice was maintained without any treatment. On Week 20, the plasma and spleen samples were collected from all the mice.

### 2.4 Identification of hUC‐MSCs

The hUC‐MSCs (Shandong Qilu Cell Therapy Engineering Technology, China) from healthy volunteers were cultured in serum‐free S&XFM complete medium (Jing‐Meng Stem Cell, China). The study used passages 3–5 hUC‐MSCs. Flow cytometry was performed to identify hUC‐MSCs markers CD34, CD45, CD73, CD90, CD105, and HLA‐DR (all antibodies were purchased from BD Bioscience, USA). Adipocytes, osteocytes, and chondrocytes were induced following coculturing hUC‐MSCs with corresponding differentiation culture media (Jing‐Meng Stem Cell, China), and cell type was identified by oil red o, alizarin red, and alcian blue staining, respectively.

### 2.5 Human CD4^+^ T Cell Isolation, Stimulation and Coculture

Human peripheral blood mononuclear cells were separated by density gradient centrifugation. CD4^+^ T cells isolated by anti‐human CD4 particles‐DM (50 µL of particles for every 10^7^ cells, BD Biosciences) were cultured in the apical chamber of transwell plate (0.4 µm; Corning Costar, USA) pre‐coated with 10 μg/mL anti‐CD3 (Leinco Technologies Inc., USA). For the hUC‐MSCs treatment (MSCT) group, the fifth passage of hUC‐MSCs were used for 72 h coculturing with CD4^+^ T cells (5 × 10^5^ cells) from moderate and severe SLE (SLE‐MS) groups in the basolateral chamber of transwell plate at ratios of 1: 25 (T1 group), 1: 10 (T2 group), and 1: 5 (T3 group) in complete RPMI 1640 medium supplemented with 5 μg/mL anti‐CD28 (Leinco Technologies Inc, USA). For the untreated groups, the CD4^+^ T cells from SLE patiens and HCs were cultured alone in other corresponding cell plates containing complete medium. Glucose metabolism was inhibited with 1 mM 2‐deoxy‐D‐glucose (2‐DG) and 5 mM metformin hydrochloride (Sigma‐Aldrich, USA).

### 2.6 Evaluation of Disease Activity of MRL/lpr Mice

Spot urine samples were collected at the same time each week starting from Week 10. Proteinuria was assessed using the Bradford Protein Assay Kit (XY‐Bioscience, China) according to the manufacturer's manual. The Mouse Anti‐Double Stranded DNA Antibody IgG ELISA Kit (Shanghai Bohu Biological Technology Co. Ltd., China) was used to determine anti‐ dsDNA antibody titer in all mice at 20 weeks of age.

### 2.7 Mice Splenic CD4^+^ T Cell Isolation, Stimulation, Coculture, and Treatment

MRL/lpr mice and C57BL/6 mice were euthanized at 20 weeks age, and the spleens were removed aseptically. The spleens were gently ground using a syringe plunger and passed through a 70 μm cell sieve. Spleen mononuclear cells were isolated by density gradient centrifugation with mouse Ficoll, following the manufacturer's instructions.

Splenic CD4^+^ T cells were isolated by anti‐mouse CD4 magnetic particles‐DM (50 µL of particles for every 10^7^ cells, BD Biosciences). To further explore the role of HSP90AA1, CD4^+^ T cells were pretreated with 17‐AAG. For in vitro animal experiments, splenic CD4^+^ T cells from MRL/lpr mice were randomly divided into three groups: lprT + MSCs group, lprT + 17‐AAG group and lpr T group. In lprT + MSCs group, splenic CD4^+^ T cells were cocultured with the fifth passage hUC‐MSCs at a ratio of 10:1 using transwell cell plates and stimulated with anti‐CD3 and anti‐CD28 as done in lupus patients. In lprT + 17‐AAG group, splenic CD4^+^ T cells were pretreated with heat shock protein 90 family (HSP90) inhibitor 17‐AAG (250 nM) for 6 h. lprT group served as disease control while the C57BL/6 group acted as normal control. Additionally, we established a separate group of hUC‐MSCs cultured alone. All groups were cultured in vitro for 72 h.

### 2.8 Cell Counting Kit‐8

After stimulation or coculturing with different ratios of hUC‐MSCs, the viability of CD4^+^ T cell was measured with cell counting kit‐8 (CCK‐8; Share‐bio, China) according to the manufacture's manual [25].

### 2.9 Cytokine Detection

Cytokine expression was measured by Luminex liquid chip technology according to the instructions of the Bio‐Plex pro human cytokine 48‐plex screening panel (Bio‐RAD, USA).

### 2.10 Extracellular Metabolic Flux Analysis

CD4^+^ T cells (5 × 10^5^ per well) were seeded in a Poly‐D‐lysine Hydrobromide (BD Biosciences, USA) pre‐coated plate, supplemented with Seahorse XF RPMI medium (Agilent Technologies, CA). The oxygen consumption rate (OCR) and the extracellular acidification rate (ECAR) were determined by Agilent Seahorse XF24 analyzer using Mito Stress Test Kit and Glycolytic Rate Assay Kit (Catalog# 103015‐100, 103344‐100; Agilent Technologies, CA).

### 2.11 Transcriptome Sequencing

RNA was extracted using Trizol Reagent (Invitrogen, Carlsbad, CA). RNA integrity was assessed using RNA Nano 6000 Assay Kit of the Bioanalyzer 2100 System (Agilent Technologies, CA). The RNA‐seq libraries were created on acBot Cluster Generation System by TruSeq PE Cluster Kitv3‐cBot‐HS (Illumina, USA). RNA sequencing was analyzed using Illumina NovaSeq. 6000 platform (Illumina, USA). *P*‐value < 0.05 and | log_2_ fold change | > 1 were considered as significantly different expression.

### 2.12 Flow Cytometry Analysis

Phenotypic cell analyses were conducted by co‐staining peripheral blood mononuclear cells with fluorochrome‐labeled monoclonal antibodies (mAbs) targeting CD4, CD25, IFN‐γ, IL‐4, IL‐17, and Foxp3 following standard protocols. All mAbs were obtained from Beckman Coulter and Becton Dickinson, USA. The cells were analyzed using NAVIOS flow cytometer (Beckman Coulter, USA) with Kaluza software for data analysis.

### 2.13 Real‐Time Quantitative PCR Analysis

First‐strand cDNA was synthesized using SweScript all‐in‐one SuperMix (Servicebio Technology, G3337, China). SYBR Green Master Mix (Servicebio Technology, China) and Applied Nanodrop 2000 real‐time PCR systems were used to detect gene expression. The relative expression of gene was normalized to human β‐actin and measured using the △*C_q_* method. The primer list is displayed in Table S1.

### 2.14 Western blot Analysis

CD4^+^ T cells were washed twice with PBS and lysed. Protein samples were extracted and separated on a 10% precast Bis‐Tris gel (Thermofisher Scientific). The separated protein was then transferred into polyvinylidene difluoride (PVDF, Millipore, USA) and blocked with 5% nonfat milk for 1 h at room temperature. The transferred PVDF membranes were incubated overnight at 4°C with primary antibodies, followed by incubation with appropriate secondary antibodies for 1 h at room temperature, in accordance with standard procedures. Primary antibodies used included anti‐HSP90AA1, anti‐PI3K, anti‐AKT, anti‐phosphorylated AKT, anti‐GLUT1, and anti‐LDHA (all diluted 1:1000; HUABIO, China). Enhanced chemiluminescence technique was used to detect protein bands, which were visualized using the Chemi Scope 4300Pro Luminescent Image Analyzer (Clinx, China).

### 2.15 Statistical Analysis

Quantitative data with normal distribution were presented as mean ± standard deviation (SD), and were analyzed by two‐tailed *t*‐test or one‐way analysis of variance (ANOVA) with multiple test correction. Welch's ANOVA or Mann–Whitney test was used to compare non‐normally distributed data. The statistical analysis was performed using GraphPad Prism (v.9) software. The classification of statistical significance was determined as: *p < 0.05; **p < 0.01; ***p < 0.001; ****p < 0.0001.

## 3 Results

### 3.1 Characteristics of Study Subjects

**Figure 1:**
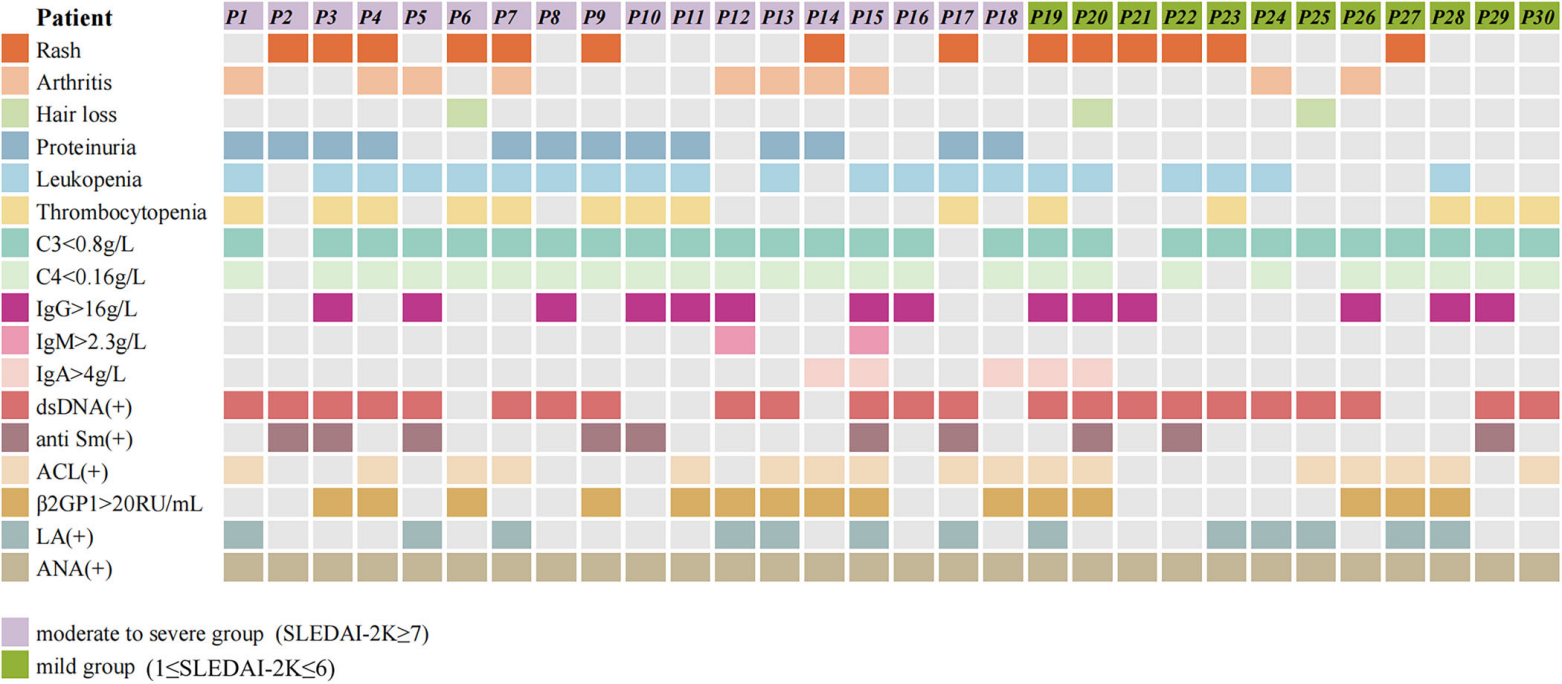
Clinical base line of lupus patients. ACL, antiphospholipid antibody; ANA, antinuclear antibody; C3, complement factor 3; C4, complement factor 4; dsDNA, double‐strand DNA; LA, lupus anticoagulants; SLEDAI‐2K, Systemic Lupus Erythematosus Disease Activity Index‐2000.

The SLE group enrolled 30 patients with disease duration less than 12 months; 3 men and 27 women, with a mean age of 32.7 ± 11.9 years, and BMI 20.67 ± 3.27 kg/m^2^. The clinical base line of lupus patients was displayed in Figure 1. The healthy controls (HCs) group enrolled 20 age, sex, and BMI matched healthy volunteers, 4 men and 16 women, with a mean age of 31.9 ± 4.8 years, and BMI 21.01 ± 2.86 kg/m^2^ (all *p* < 0.05).

### 3.2 Identification of hUC‐MSCs

**Figure 2:**
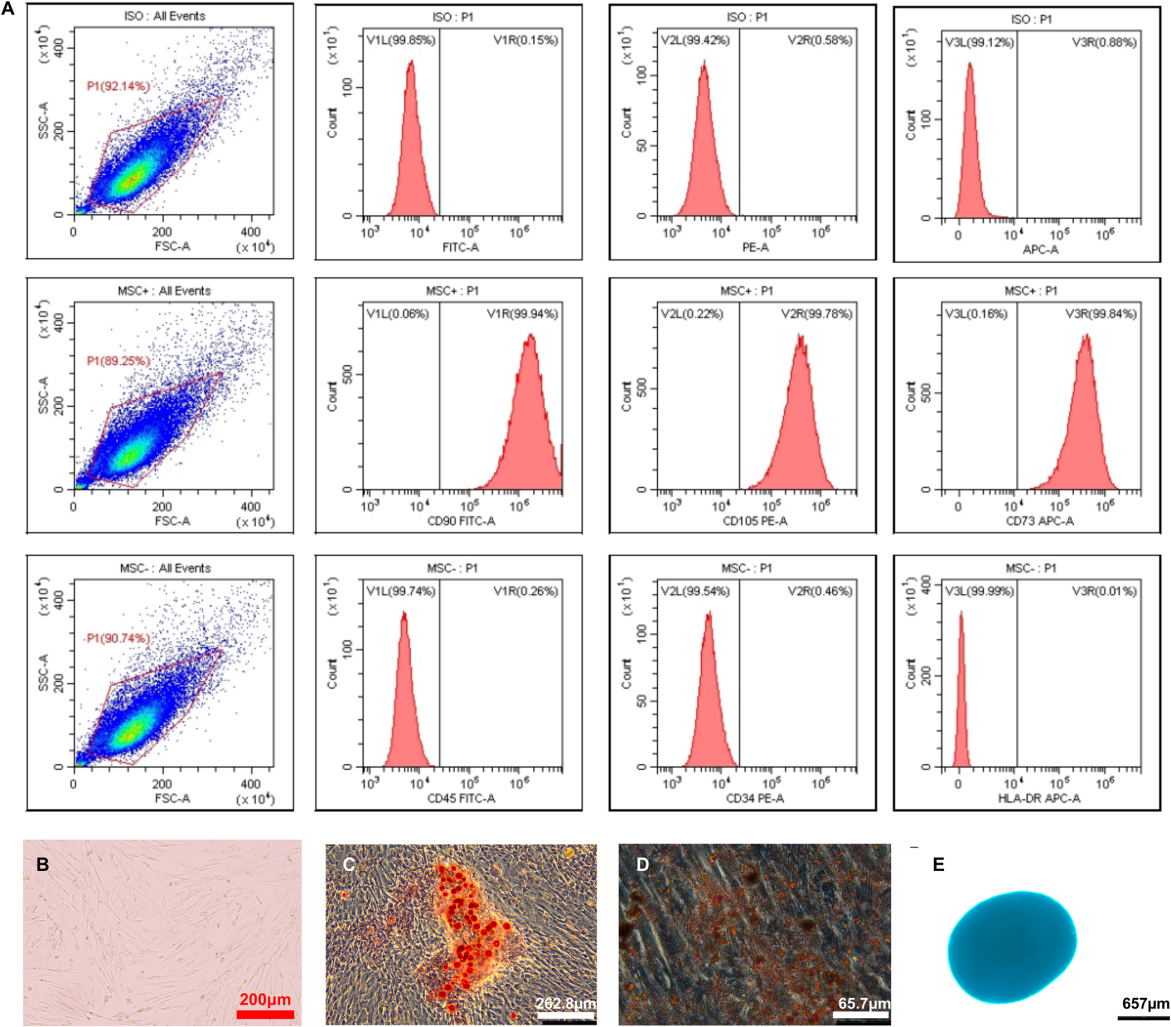
Identification and differentiation of hUC‐MSCs. (A) The expression of CD90, CD105, and CD73 were 99.94%, 99.78%, and 99.84%, respectively. The expression of CD45, CD34, and HLA‐DR were 0.26%, 0.46%, and 0.01%, respectively. (B) hUC‐MSCs showed spindle form such as fibroblast‐like cells in vitro culture (×100 magnification; scale bar, 200 μm). (C–E) The differentiate capacity of hUC‐MSCs was analyzed by culturing the cells in a differentiation medium for 3–4 weeks. After incubation, the osteocytes, adipocytes and chondrocytes were visualized as red color after alizarin red (C) and oil red o staining (D), blue color after alcian blue staining (E), respectively (magnification, ×100, ×400, and ×40, respectively; scale bar, 262.8, 65.7, and 657 μm, respectively).

As shown in Figure 2A, flow cytometry showed high expression of CD73, CD90, CD105, and no or low levels of CD34, CD45, and HLA‐DR, and conformed the standard of the International Society for Cellular Therapy for hUC‐MSC. The fifth passage of hUC‐MSCs with serum‐free complete medium exhibited typically monolayered spindle‐shaped cells with capability of adhering to cell plates (Figure 2B). To induce osteogenic, adipogenic, and chondrogenic differentiation, hUC‐MSCs were cultured in the respective differentiation medium for a duration of 3 weeks. The hUC‐MSCs subjected to osteogenic differentiation medium exhibited dense red opaque areas as revealed by alizarin red staining. The hUC‐MSCs cultured with adipogenic differentiation medium displayed numerous oil droplets within the cytoplasm, as indicated by oil red O staining. Furthermore, hUC‐MSCs exposed to chondrogenic differentiation medium demonstrated typical light blue chondrogenic pellets following alcian blue staining (Figure 2C–E). After coculturing with CD4^+^ T cells, the flow cytometry results and cell morphology of hUC‐MSCs were shown in Figure S1.

### 3.3 Treatment With hUC‐MSCs Downregulated the CD4^+^ T Cell Glucose Metabolism in Lupus

#### 3.3.1 The Overactive Glucose Metabolic Capacity of SLE‐CD4^+^ T Cell Was Associated With Disease Activity

**Figure 3:**
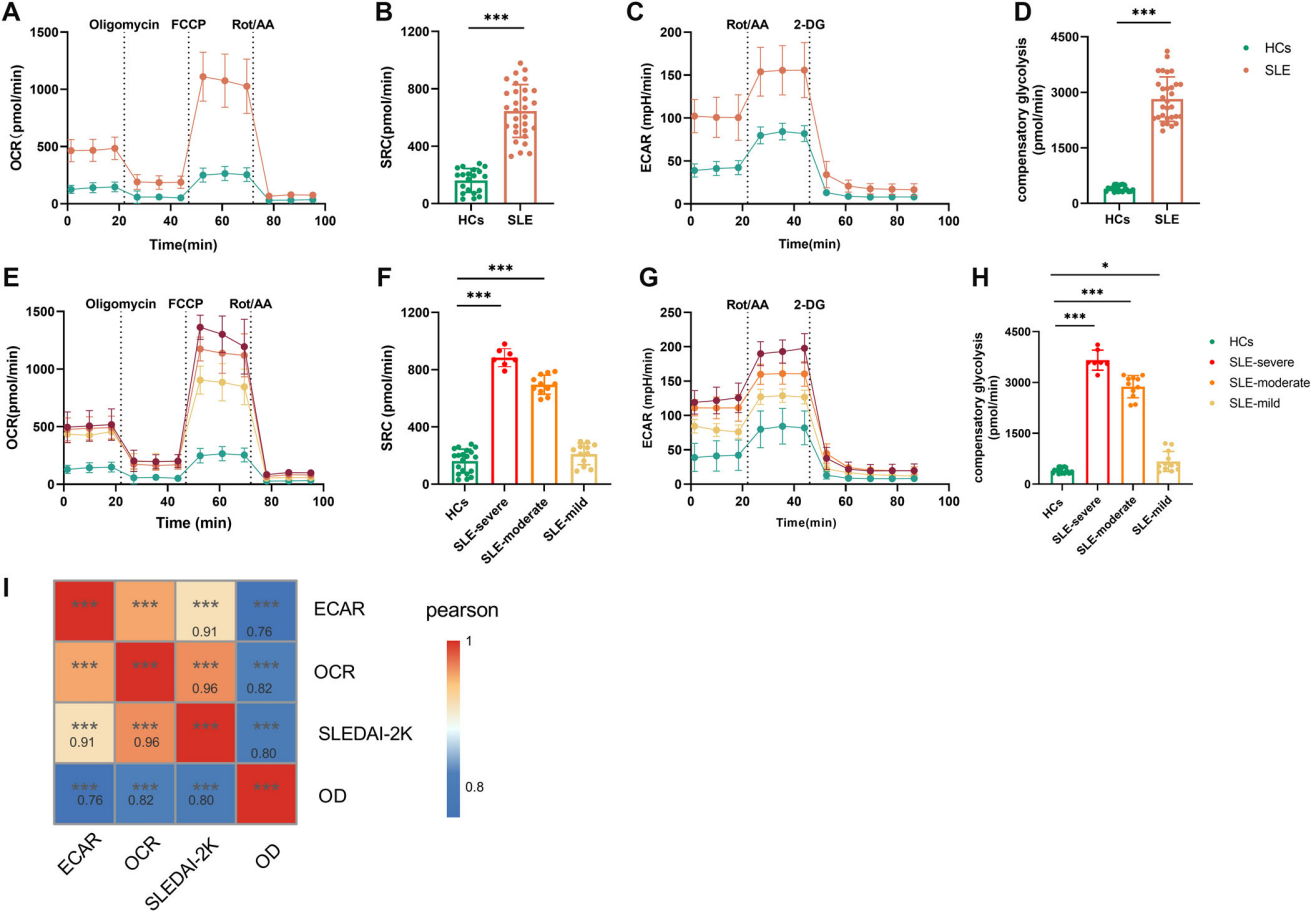
Glucose metabolism analysis of CD4^+^ T cells in lupus patients. (A–D) The OCR (A), spare respiration capacity (B), ECAR (C), and compensatory glycolysis (D) of CD4^+^ T cells in SLE group and controls. (E–H) The OCR (E), spare respiration capacity (F), ECAR (G), and compensatory glycolysis (H) of CD4^+^ T cells in SLE patients with different activity and controls. (I) The correlation between glucose metabolism and expansion of CD4^+^ T cells, and disease activity in SLE. SLE group, *n* = 30; SLE‐severe group, *n* = 7; SLE‐moderate group, *n* = 11; SLE‐mild group, *n* = 12; HCs, *n* = 20. 2‐DG, 2‐deoxyglucos; ECAR, extracellular acidification rate; FCCP, mitochondrial oxidative phosphorylation uncouple carbonyl cyanide 4‐trifluoromethoxy phenylhydrazone; HCs, healthy controls; OCR, oxygen consumption rate; OD, optical density; Rot/AA, rotenone/antimycin A; SRC, spare respiration capacity. *p < 0.05, ***p < 0.001.

Recent research has highlighted the mutual influence of cellular metabolism and immune inflammatory response. To evaluate the impact of glucose metabolic reprogramming in patients with SLE, we initially assessed the oxidative phosphorylation and glycolysis profiles of activated SLE‐CD4^+^ T cell through real‐time metabolic analysis utilizing Agilent seahorse XF24 analyzer. Mitochondrial oxidative phosphorylation was quantified by measuring OCR. Spare respiratory capacity (SRC) serves as a quantitative metric for mitochondrial function via oxidative phosphorylation. The level of glycolysis was determined by ECAR. Compensatory glycolysis is a measurable parameter employed to estimate glycolytic activity. Extracellular metabolic flux analyses showed significantly upregulated mitochondrial respiration in CD4^+^ T cells from SLE group compared with HCs, as indicated by increased SRC, basal respiration, maximal respiration, proton leak, and ATP production (Figures 3A,B and S3A–D). The aerobic glycolysis was also enhanced with increased compensatory glycolysis, basal glycolysis, and basal proton efflux rate (Figures 3C,D and S4A,B).

To further measure the correlation between glucose metabolism activation and disease activity, we analyzed the mitochondrial oxidative phosphorylation and aerobic glycolysis capacity in different disease states. As shown in Figure 3E,F, SRC of CD4^+^ T cells in moderate and severe SLE groups (695.72 ± 63.88 and 884.31 ± 57.69 pmol/min) was significantly increased compared with HCs (161.54 ± 80.83 pmol/min, both *p* < 0.001), as well as basal respiration, maximal respiration, proton leak, and ATP production (Figure S3E–H). Compensatory glycolysis of CD4^+^ T cells in mild, moderate, and severe SLE groups (668.77 ± 283.97, 2874.94 ± 312.06, and 3657.59 ± 273.23 pmol/min) was significantly increased compared with controls (401.11 ± 77.76 pmol/min, all *p* < 0.05; Figure 3G,H), as well as basal glycolysis and basal proton efflux rate (Figure S4C,D). Notably, the glucose metabolism of CD4^+^ T cells in SLE‐MS groups was strongly positively correlated with SLEDAI‐2K (both *p* < 0.001; *r* = 0.91 and 0.96) and proliferation capacity (both *p* < 0.001; *r* = 0.76 and 0.82) (Figure 3I).

#### 3.3.2 Treatment With hUC‐MSCs Downregulated the CD4^+^ T Cell Glycolysis and Oxidative Phosphorylation of SLE

**Figure 4:**
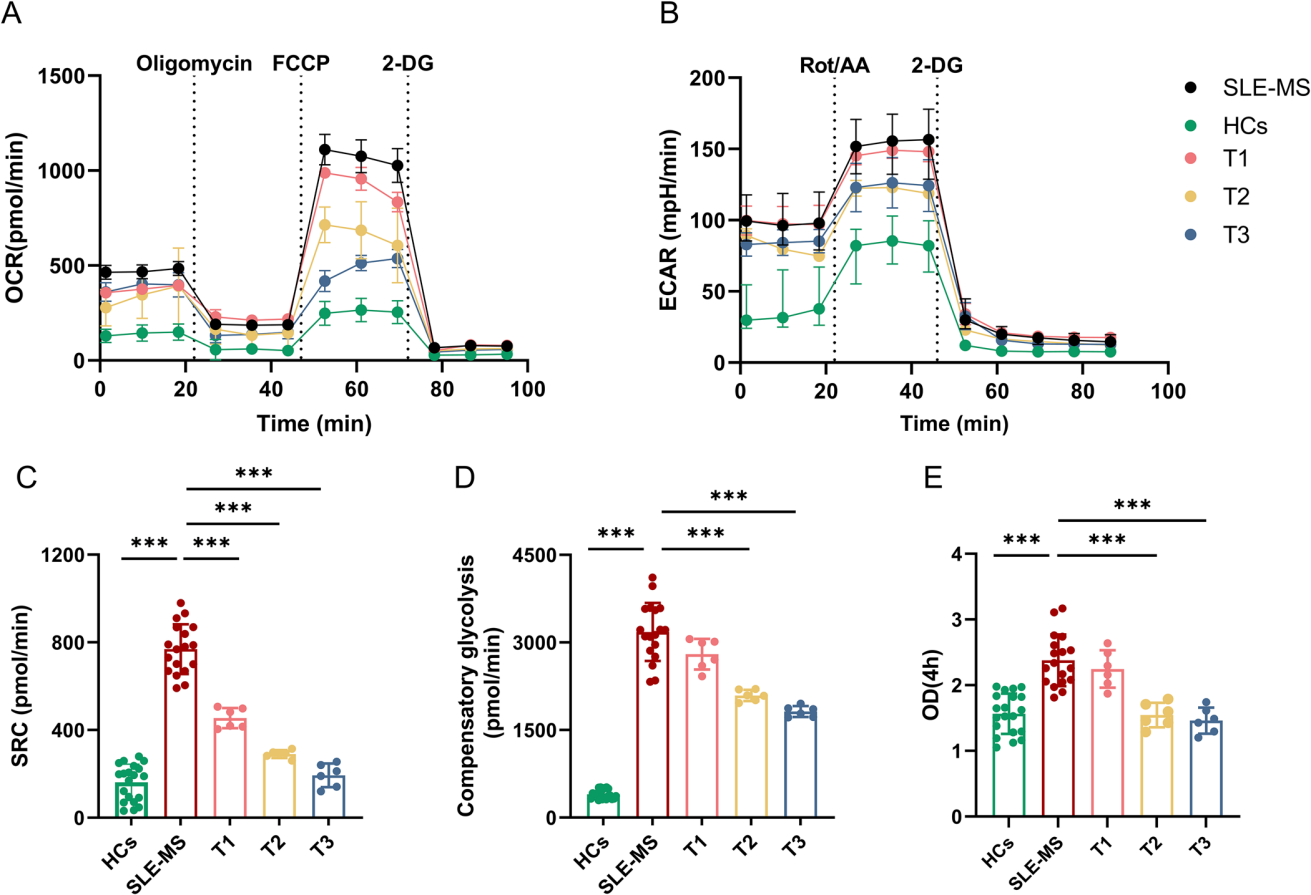
The hUC‐MSCs treatment effects on glycometabolic capacity and proliferation of CD4^+^ T cell in lupus patients with moderate and severe activity. The OCR (A) and ECAR (B) of CD4^+^ T cells from SLE‐MS group, MSCT groups, and HCs. (C–E) The spare respiratory capacity (C), compensatory glycolysis (D) and OD (E) of CD4^+^ T cells in a fixed number cocultured with hUC‐MSCs at different ratios in SLE‐MS group. HCs, *n* = 20; SLE‐MS group, *n* = 18; T1, T2, and T3 groups, *n* = 6; respectively. 2‐DG, 2‐deoxyglucos; ECAR, extracellular acidification rate; FCCP, mitochondrial oxidative phosphorylation uncouple carbonyl cyanide 4‐ trifluoromethoxy phenylhydrazone; HCs, healthy controls; MSCT, hUC‐MSCs treatment; OCR, oxygen consumption rate; OD optical density; Rot/AA, rotenone/antimycin A; SLE‐MS, patients with moderate and severe SLE; SRC, spare respiration capacity. ***p < 0.001.

As shown above, CD4^+^ T cells from SLE‐MS group had strongly enhanced glucose metabolic activation, compared with HCs (Figure 3F,H). By contrast, we observed no significant differences in OCR between the mild group and controls (Figure 3F). Additionally, MSCs treatment primarily targets patients with advanced and refractory lupus. To minimize potential confounding factors, we cocultured hUC‐MSCs with a fixed numbers of SLE‐CD4^+^ T cell at ratios of 1:25 (T1 group), 1:10 (T2 group), and 1:5 (T3 group). The CD4^+^ T cells in T1, T2, and T3 groups were isolated from the same six patients in SLE‐MS group. After 72 h of cocultured with hUC‐MSCs, the glucose metabolic capacity of CD4^+^ T cells was inhibited. To assess mitochondrial oxidative capacity, we observed that the SRC of T1, T2, and T3 groups (454.37 ± 42.08, 290.76 ± 16.54, and 193.63 ± 49.06 pmol/min, respectively) were significantly decreased (all *p* < 0.001) compared with the SLE‐MS group (769.06 ± 110.63 pmol/min) (Figure 4A,C), as well as basal respiration, maximal respiration, proton leak, and ATP production (Figure S5A–D). For aerobic glycolytic capacity assessment, we observed that the compensatory glycolysis of T2 and T3 groups (2092.32 ± 87.28 and 1817.67 ± 84.75 pmol/min) had a significant decrease (both *p* < 0.001) compared with the SLE‐MS group (3179.31 ± 483.85 pmol/min), whereas no difference was observed with the T1 group (2798.36 ± 239.15 pmol/min) (Figure 4B,D). Moreover, we found that hUC‐MSCs significantly downregulated the basal glycolysis and basal proton efflux rate in the CD4^+^ T cells of lupus patients with moderate and severe disease activity (Figure S5E,F).

### 3.4 Treatment With hUC‐MSCs Downregulated the Proliferation Capacity of CD4^+^ T Cells in Lupus

#### 3.4.1 Overactive Glucose Metabolism Was Involved in the Upregulated Proliferation Capacity of SLE‐CD4^+^ T Cell

To delineate the regulation of hUC‐MSCs treatment on CD4^+^ T cell expansion in SLE, we first measured the OD of the CD4^+^ T cells between SLE‐MS group and HCs. As shown in Figure 4E, the OD in SLE‐MS group was significantly increased compared with HCs (2.39 ± 0.39 vs. 1.56 ± 0.29, *p* < 0.001). Notably, the OD of CD4^+^ T cells was positively correlated with ECAR and OCR in SLE‐MS group (both *p* < 0.001; *r* = 0.76 and 0.82) (Figure 3I).

#### 3.4.2 Treatment With hUC‐MSCs Downregulated the CD4^+^ T Cell Proliferation Capacity of SLE

After 72 h of coculture with different ratios of hUC‐MSCs, we observed that the ODs in the T2 and T3 groups were significantly decreased compared with the SLE‐MS group (1.70 ± 0.23 vs. 2.39 ± 0.39 and 1.65 ± 0.23 vs. 2.39 ± 0.39, both *p* < 0.001), whereas no difference was observed between T1 group and the SLE‐MS group (2.19 ± 0.27 vs. 2.39 ± 0.39) (Figure 4E).

### 3.5 Treatment With hUC‐MSCs Modulated the Inflammatory Factors Secreted by CD4^+^ T Cells in Lupus

#### 3.5.1 Treatment With hUC‐MSCs Decreased Supernatant Interferon (IFN)‐γ Concentration

**Figure 5:**
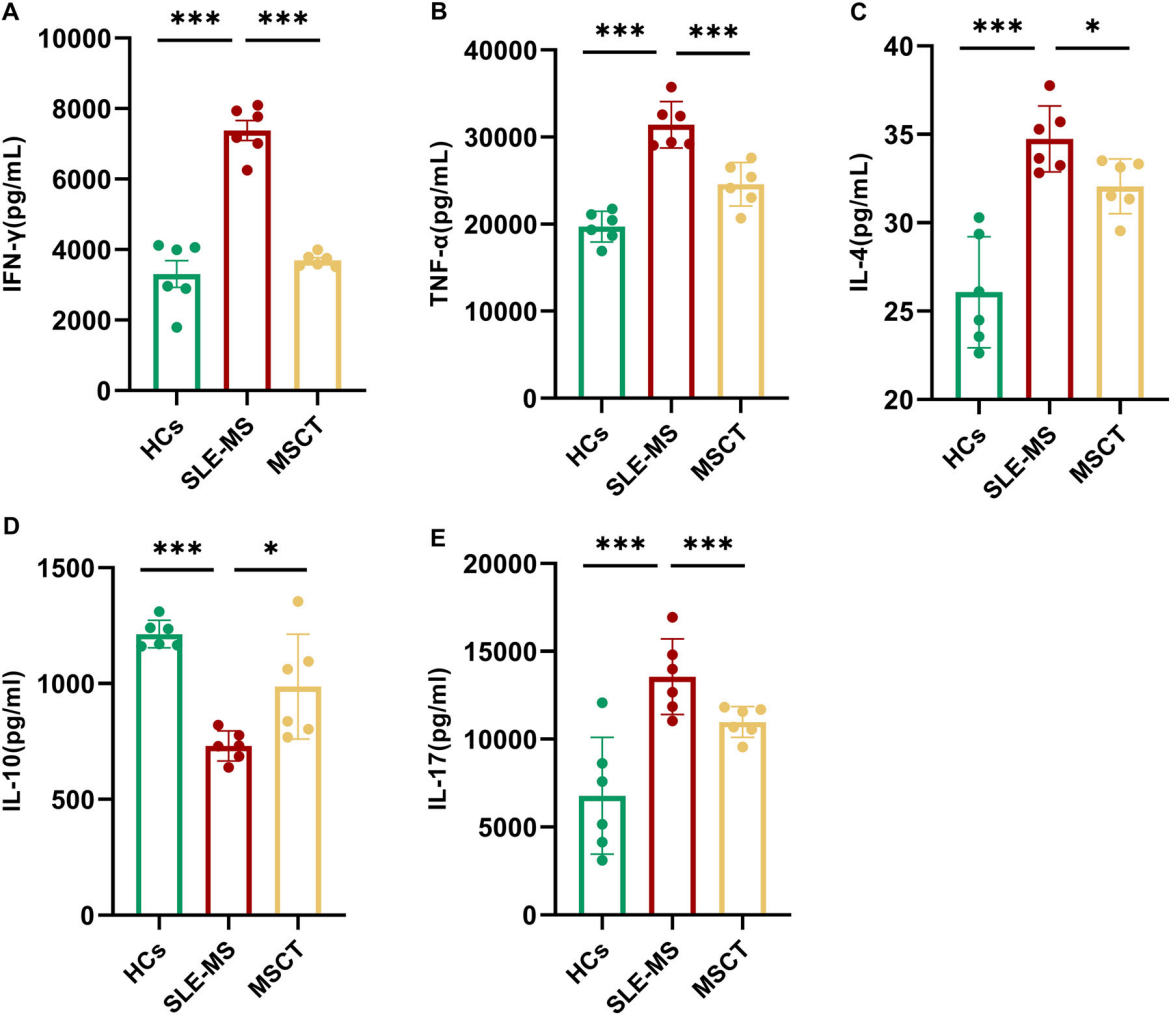
The hUC‐MSCs treatment effects on CD4^+^ T cell cytokine secretion in lupus patients with moderate and severe activity. (A–E) Treatment with hUC‐MSCs significantly decreased the supernatant levels of IFN‐γ (A), TNF‐α (B), IL‐4 (C), and IL‐17 (E), while increased IL‐10 level (D). SLE‐MS group, patients with moderate and severe SLE, *n* = 6; MSCT group, CD4^+^ T cells from SLE‐MS group cocultured with hUC‐MSCs at the ratio of 10: 1, *n* = 6; HCs, healthy controls, *n* = 6. IFN‐γ, interferon γ; IL, interleukins; TNF‐α, tumor necrosis factor‐α. *p < 0.05, ***p < 0.001.

To investigated the effects of hUC‐MSCs on cytokine secretion of CD4^+^ T cell, we measured the supernatant cytokine concentrations in SLE‐MS group (*n* = 6) with or without hUC‐MSCs coculturing and in the HCs (*n* = 6) (Table S2). As shown in Figure 5A, SLE‐MS group exhibited a higher expression of IFN‐γ in the supernatant of CD4^+^ T cells (7376 ± 587.88 pg/mL), compared with HCs (3306 ± 782.91 pg/mL; *p* < 0.001). The hUC‐MSCs significantly reduced the IFN‐γ concentration in the supernatant (3698.27 ± 155.15 pg/mL; *p* < 0.001).

#### 3.5.2 Treatment With hUC‐MSCs Decreased the Supernatant TNF‐α Concentration

As shown in Figure 5B, we observed the supernatant TNF‐α concentation was reduced in the MSCT group, compared with the SLE‐MS group (24579.33 ± 2118.99 vs. 31407 ± 2255.22 pg/mL; *p* < 0.001). There was a significant difference between SLE‐MS group and controls (19,708 ± 1496.29 pg/mL, *p* < 0.001).

#### 3.5.3 Treatment With hUC‐MSCs Decreased the Supernatant IL‐4 Concentration

Compared with HCs, the expression of IL‐4 in the supernatant enhanced in the SLE‐MS group (34.74 ± 1.58 vs. 24.40 ± 1.04 pg/mL; *p* < 0.001). After cocultured with hUC‐MSCs, the concentration of IL‐4 was significantly decreased (32.06 ± 1.31 pg/mL; *p* < 0.05) (Figure 5C).

#### 3.5.4 Treatment With hUC‐MSCs Increased the Supernatant IL‐10 Concentration

Compared with controls, the supernatant level of IL‐10 was decreased in the SLE‐MS group (730.33 ± 54.69 vs. 1213.33 ± 50.12 pg/mL; *p* < 0.001). The hUC‐MSCs treatment significantly increased the concentration of IL‐10 (986.46 ± 191.31 pg/ml, *p* < 0.05) (Figure 5D).

#### 3.5.5 Treatment With hUC‐MSCs Decreased the Supernatant IL‐17 Concentration

As shown in Figure 5E, hUC‐MSCs treatment decreased IL‐17 concentration in the supernatant compared with the SLE‐MS group (10,983 ± 1013.16 vs. 13,554.33 ± 482.53 pg/mL; *p* < 0.05). In addition, we observed a significant difference between SLE‐MS group and HCs (6783.33 ± 3029.92 pg/mL, *p* < 0.001).

### 3.6 Treatment With hUC‐MSCs Modulated SLE‐CD4^+^ T Cell Inflammatory Factors Production by Reinstating Overactive Glucose Metabolism via *HSP90AA1* in the PI3K‐AKT Pathway

**Figure 6:**
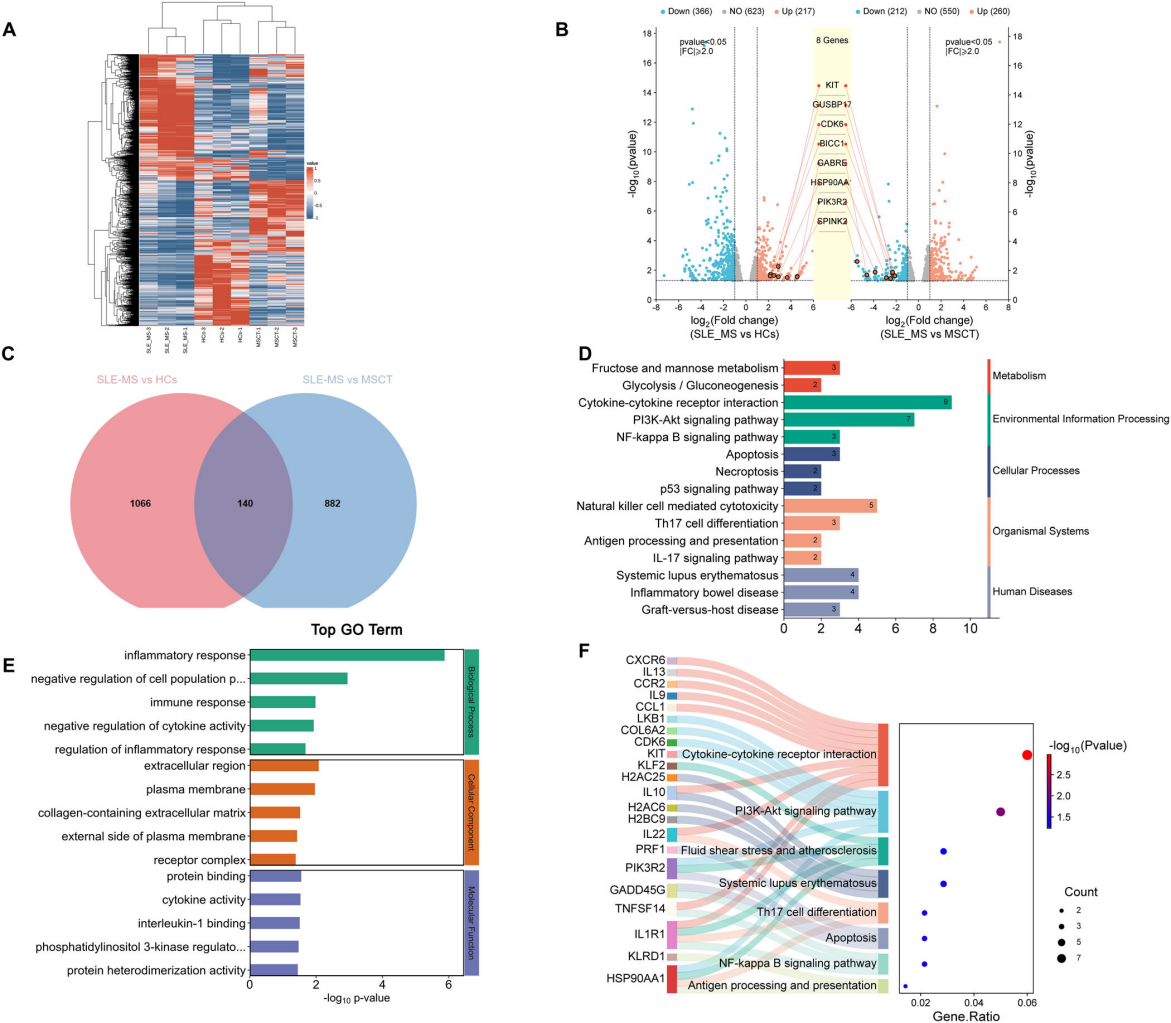
Transcriptomic analysis of CD4^+^ T cells in lupus patients with moderate and severe activity cocultured with or without hUC‐MSCs and HCs. (A) RNA sequencing shows gene expression profiling of DEGs in three groups. (B) Volcano plots of DEGs of two sets of gene clusters and the downregulated genes of MSCT group. (C) Wayne diagram of two sets of DEGs clusters. (D–F) KEGG enrichment analysis (D), GO enrichment analysis (E), and Sankey plot (F) of DEGs from the intersecting cluster. SLE‐MS group, patients with moderate and severe SLE, *n* = 3; MSCT group, CD4^+^ T cells from SLE‐MS group cocultured with hUC‐MSCs at the ratio of 10: 1, *n* = 3; HCs, healthy controls, *n* = 3. DEGs, differentially expressed genes; GO, gene ontology; KEGG, Kyoto encyclopedia of gene and genomes.

To identify the therapeutic mechanism of hUC‐MSCs on regulating inflammatory cytokines of SLE‐CD4^+^ T cell, RNA sequencing (RNA‐seq) analysis of CD4^+^ T cells was performed in the SLE‐MS group, MSCT group, and HCs (*n* = 3, respectively). The mRNA clustering showed significantly different expression of genes between the three groups (Figure 6A). We found 1206 genes differentially expressed (DEGs) between the SLE‐MS group and HCs. There were 217 DEGs significantly upregulated in the SLE‐MS group, whereas 366 were significant downregulated, compared with HCs (Figure 6B). We found 1022 DEGs between the MSCT group and SLE‐MS group; 260 DEGs were significant upregulated in the MSCT group, whereas 212 were significant downregulated, compared with the SLE‐MS group. The top eight DEGs that exhibited an increase in the SLE‐MS group and a decrease in MSCT group were *HSP90AA1, KIT, GUSBP17, CDK6, BICC1, GABRE, PIK3R2*, and *SPINK2* (Figure 6B).

**Figure 7:**
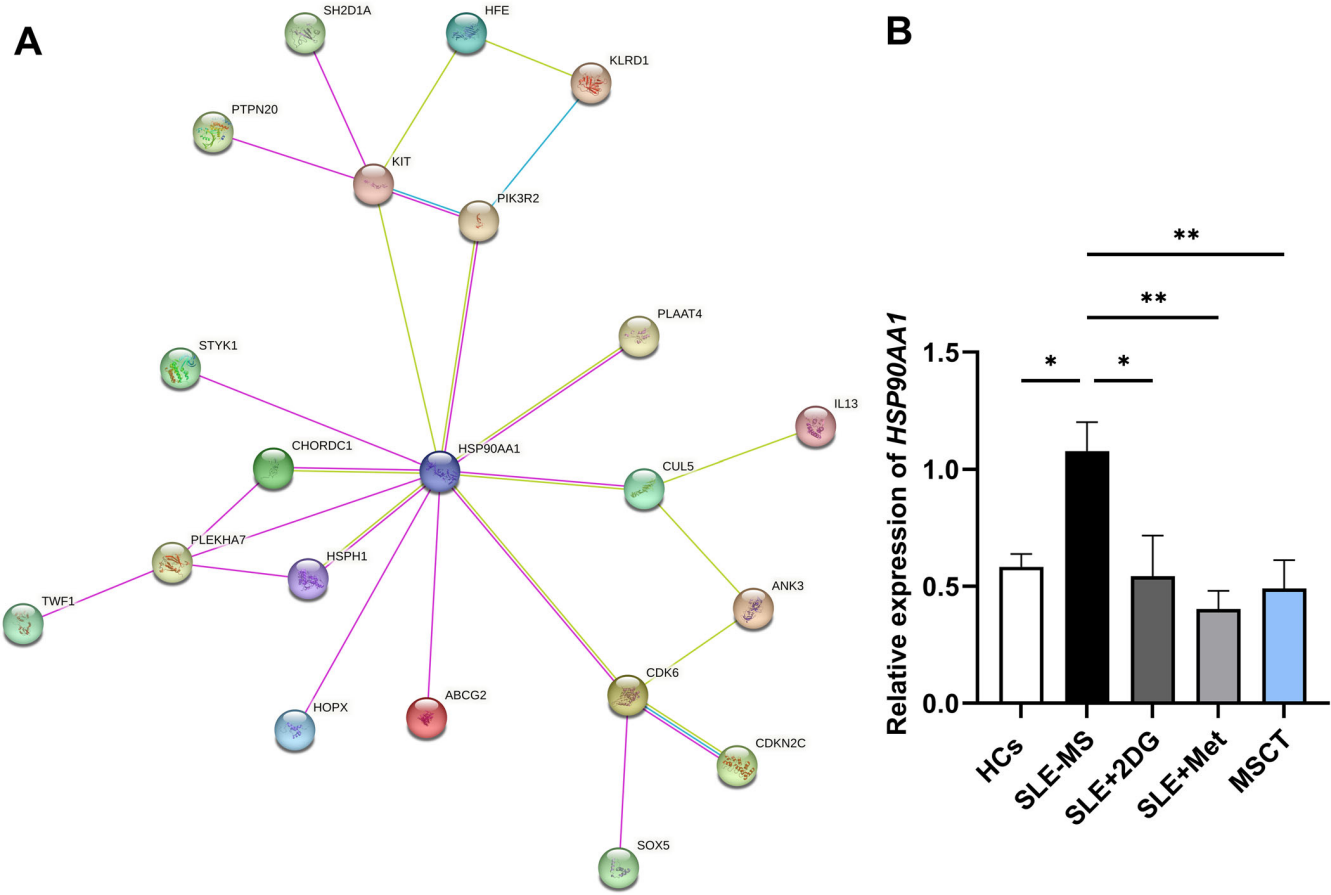
Identification of hub gene and glucose metabolism association analysis. (A) Gene interaction network analysis of DEGs from the intersecting cluster. (B) The mRNA expression of *HSP90AA1* in CD4^+^ T cells from patients with moderate and severe SLE cocultured with or without hUC‐MSCs, 2‐DG, and metformin. SLE‐MS group, patients with moderate and severe SLE, *n* = 3; MSCT group, CD4^+^ T cells from SLE‐MS group cocultured with hUC‐MSCs at the ratio of 10: 1, *n* = 3; SLE + 2‐DG group, CD4^+^ T cells from SLE‐MS group cocultured with 2‐DG at the concentration of 1 mM, *n* = 3; SLE+Met group, CD4^+^ T cells from SLE‐MS group cocultured with metformin at the concentration of 5 mM, *n* = 3; HCs, healthy controls, *n* = 3. 2‐DG, 2‐deoxyglucos; Met, metformin. *p < 0.05; **p < 0.01.

We constructed a Wayne diagram of two sets of differentially expressed mRNA. We found 140 mRNAs in the intersecting cluster of DEGs (Figure 6C). Kyoto encyclopedia of gene and genomes (KEGG) enrichment analysis revealed 15 significant pathways involving 140 mRNAs. The top two pathways in each major group include: fructose and mannose metabolism pathway (hsa00051) and glycolysis/gluconeogenesis pathway (hsa00010) for the metabolism group; PI3K‐AKT signaling pathway (hsa04151) and cytokine‐cytokine receptor interaction pathway (hsa04060) for the environment information processing group; apoptosis (hsa04210) and necroptosis (hsa04217) for the cellular processes group; natural killer cell mediated cytotoxicity (hsa04650) and Th17 cell differentiation pathway (hsa04659) for the organismal systems group; as well as SLE (hsa05322) and inflammatory bowel disease (hsa05321) for human disease group (Figure 6D). Gene ontology (GO) analysis revealed enrichment in biological process such as inflammatory response (GO:0006954), negative regulation of cell population proliferation (GO:0008285); cellular component including extracellular region (GO:0005576) and plasma membrane (GO:0005886); along with molecular function like protein binding (GO:0005515) and cytokine activity (GO:0005125) (Figure 6E). Gene interaction network analysis also indicated that *HSP90AA1* was at the center of a network, and *PIK3R2, CDK6*, and *KIT* had more clusters compared with other DEGs (Figure 7A). The PI3K‐AKT signaling pathway enriched all of these annotated genes with high interaction evidence (Figure 6F).

To investigate the role of *HSP90AA1* in the hUC‐MSCs treatment, we first detected the expression change of *HSP90AA1* in the SLE‐MS group, MSCT group, and HCs. The qRT‐PCR data revealed an increased expression of *HSP90AA1* in the SLE‐MS group compared with HCs, and a decreased expression of *HSP90AA1* after hUC‐MSCs treatment compared with the SLE‐MS group (Figure 7B). To experimentally confirm the glucose metabolic modulation of *HSP90AA1* in PI3K‐AKT signaling pathway, we used 2‐DG and metformin to inhibit glycolysis and oxidative phosphorylation of SLE‐CD4^+^ T cells, respectively, and then detected the expression change of *HSP90AA1*. As shown in Figure 7B, there was a significant decrease in the expression of *HSP90AA1* following treatment with 2‐DG and metformin, compared with SLE‐MS group.

### 3.7 Systemic Administration of hUC‐MSCs Reduced Disease Activity in Lupus‐Prone MRL/lpr Mice

**Figure 8:**
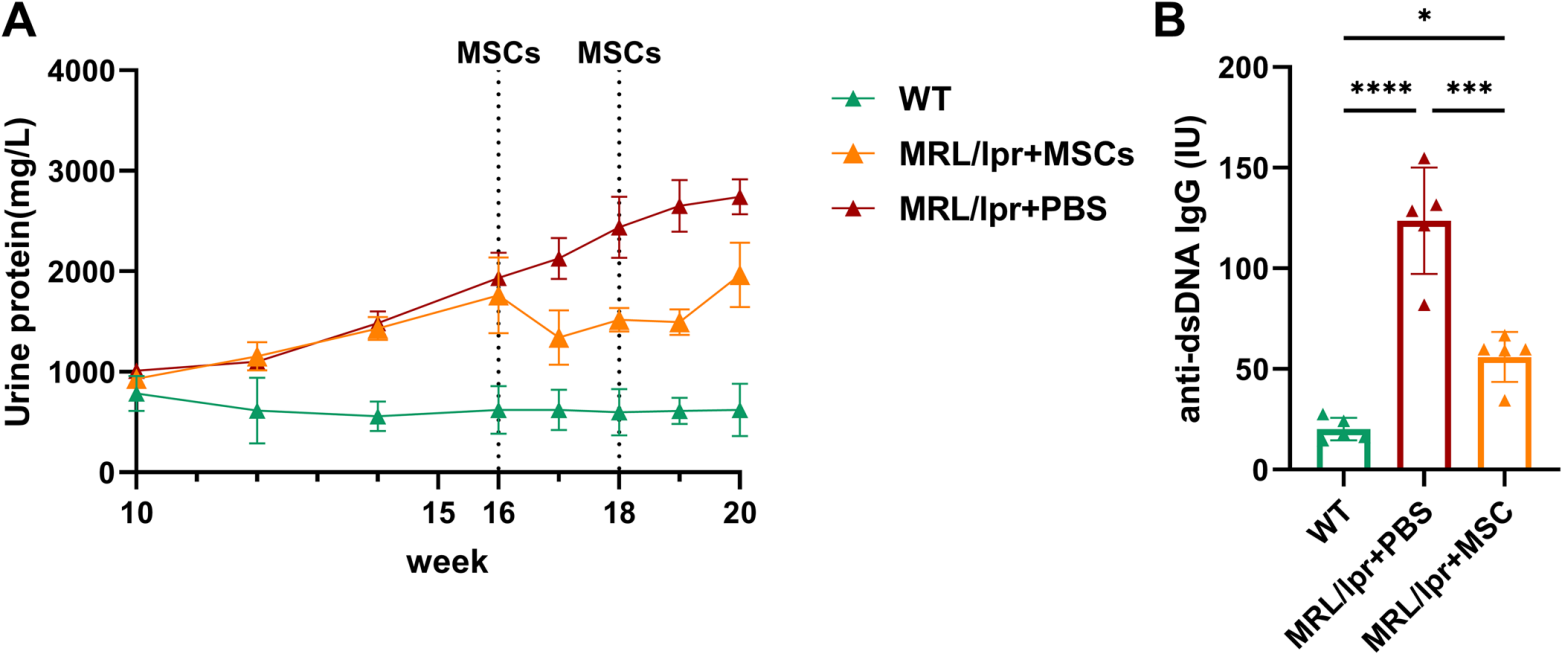
Systemic administration of hUC‐MSCs relieved MRL/lpr mice disease manifestations. (A) Changes of proteinuria from Week 10 to 20. Systemic administration of hUC‐MSCs significantly decreased the urine protein of lupus prone mice at Weeks 17–20. (B) Systemic administration of hUC‐MSCs particularly inhibited anti‐dsDNA antibodies formation in the serum of MRL/lpr mice. All *n* = 5. *p < 0.05, ***p < 0.001, ****p < 0.0001.

To evaluate the efficacy of hUC‐MSCs in SLE, we monitored proteinuria weekly and measured plasma anti‐dsDNA antibody titers in mice aged 20 weeks. The results indicated that TVI of hUC‐MSCs improved renal function in lupus prone mice. In comparison to C57BL/6 mice, the urine protein levels of MRL/lpr mice exhibited a significant and progressive increase with advancing weeks of age. Treatment with hUC‐MSCs resulted in a reduction of proteinuria in MRL/lpr + MSCs group compared with MRL/lpr + PBS group from 17 to 20 weeks (Figure 8A and Table 1). As shown in Figure 8B, the plasma levels of anti‐dsDNA antibody in MRL/lpr mice were significantly elevated compared to normal controls (123.65 ± 23.68 vs. 20.12 ± 4.96 IU, *p* < 0.0001). Administration of hUC‐MSCs via TVI significantly decreased the plasma antibody titer in MRL/lpr + MSCs group compared to disease controls group in 20 weeks (55.97 ± 11.14 vs. 123.65 ± 23.68 IU, *p* < 0.001). The results provided evidence that hUC‐MSCs can alleviate disease activity in lupus‐prone mice.

**Table 1:** Systemic administration of hUC‐MSCs exhibited a significant reduction of proteinuria levels in MRL/lpr mice.

Mice age	WT	MRL/lpr+PBS	MRL/lpr+MSCs
(Week)	Mean (SD) *n* = 5	Mean (SD) *n* = 5	Mean (SD) *n* = 5
10	784.05 (156.24)	1012.74 (60.30)	932.09 (68.30)
12	614.36 (291.28)	1102.54 (43.50)	1154.30 (125.82)
14	558.53 (130.40)	1484.89 (105.81)^####^	1432.61 (103.27)
16	622.67 (212.79)	1933.81 (225.40)^####^	1762.67 (336.91)
17	621.16 (179.17)	2127.28 (183.54)^####^	1341.16 (243.06)**
18	599.10 (205.05)	2439.43 (272.57)^####^	1518.99 (104.51)**
19	613.09 (118.00)	2651.87 (231.09)^####^	1496.48 (113.88)***
20	620.54 (234.82)	2740.14 (156.42)^####^	1966.36 (288.06)**

*Note:* For comparison between WT and MRL/lpr + PBS group, ^####^ *p* < 0.0001. For comparison between MRL/lpr + PBS and MRL/lpr + MSCs group, **p < 0.01, ***p < 0.001.

### 3.8 Systemic Administration of hUC‐MSCs Inhibited Pro‐Inflammatory Cytokines Secretion in Lupus‐Prone MRL/lpr Mice

**Figure 9:**
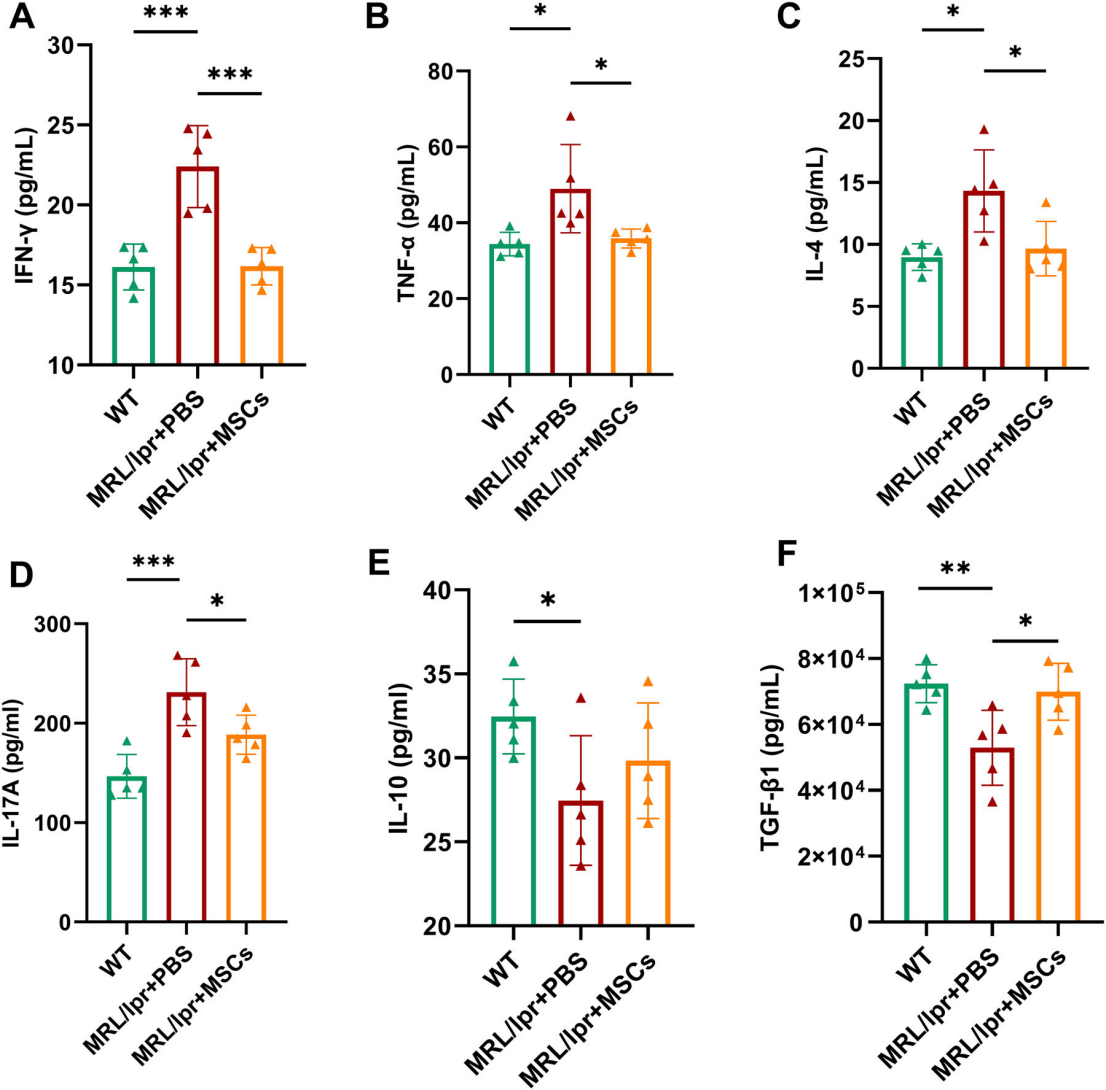
Systemic administration of hUC‐MSCs revised pro‐inflammatory profile in MRL/lpr mice. Treatment with hUC‐MSCs inhibited the serum levels of (A) IFN‐γ, (B) TNF‐α, (C) IL‐4, and (D) IL‐17A, and increased the expression of (E) IL‐10 and (F) TGF‐β1. All *n* = 5.*p < 0.05, **p < 0.01, ***p < 0.001.

To further investigated the regulation of hUC‐MSCs on inflammatory microenvironment in SLE, we measured serum cytokines levels of mice at 20 weeks of age. In MRL/lpr mice, we observed that the serum levels of IFN‐γ (22.40 ± 2.29 vs. 16.13 ± 1.28 pg/mL, *p* = 0.0004), TNF‐α (48.99 ± 10.39 vs. 34.37 ± 2.78 pg/mL, *p* = 0.02), IL‐4 (14.33 ± 2.97 vs. 8.98 ± 0.96 pg/mL, *p* = 0.01), and IL‐17A (231.21 ± 30.06 vs. 146.58 ± 19.72 pg/mL, *p* = 0.0006) were significantly increased (Figure 9A–D), while the concentration of IL‐10 (27.47 ± 3.45 vs. 32.47 ± 1.99 pg/mL, *p* = 0.03) and TGF‐β1 (52,920.39 ± 10,189.91 vs. 72,374.10 ± 5177.01 pg/mL, *p* = 0.009) were notably decreased compared with normal controls (Figure 9E,F). Intravenous administration of hUC‐MSCs inhibited the serum levels of IFN‐γ (16.18 ± 1.04 vs. 22.40 ± 2.29 pg/mL, *p* = 0.0004), TNF‐α (35.88 ± 2.24 vs. 48.99 ± 10.39 pg/mL, *p* = 0.03), IL‐4 (9.67 ± 1.97 vs. 14.33 ± 2.97 pg/mL, *p* = 0.03), and IL‐17A (188.53 ± 17.59 vs. 231.21 ± 30.06 pg/mL, *p* = 0.03) in MRL/lpr+MSCs group compared to disease control group (Figure 9A–D). Conversely, it was observed that hUC‐MSCs upregulated the level of TGF‐β1 (69,929.64 ± 7711.69 vs. 52,920.39 ± 10,189.91 pg/mL, *p* = 0.02, Figure 9F). However, no significant change in IL‐10 level was detected in MRL/lpr mice treated with hUC‐MSCs compared with disease control group (29.83 ± 3.08 vs. 27.47 ± 3.45 pg/mL, *p* = 0.34, Figure 9E).

### 3.9 Systemic Administration of hUC‐MSCs Decreased Glucose Metabolism, As Well As HSP90AA1/PI3K/AKT Signaling Pathway Activity in Lupus Prone Mice

**Figure 10:**
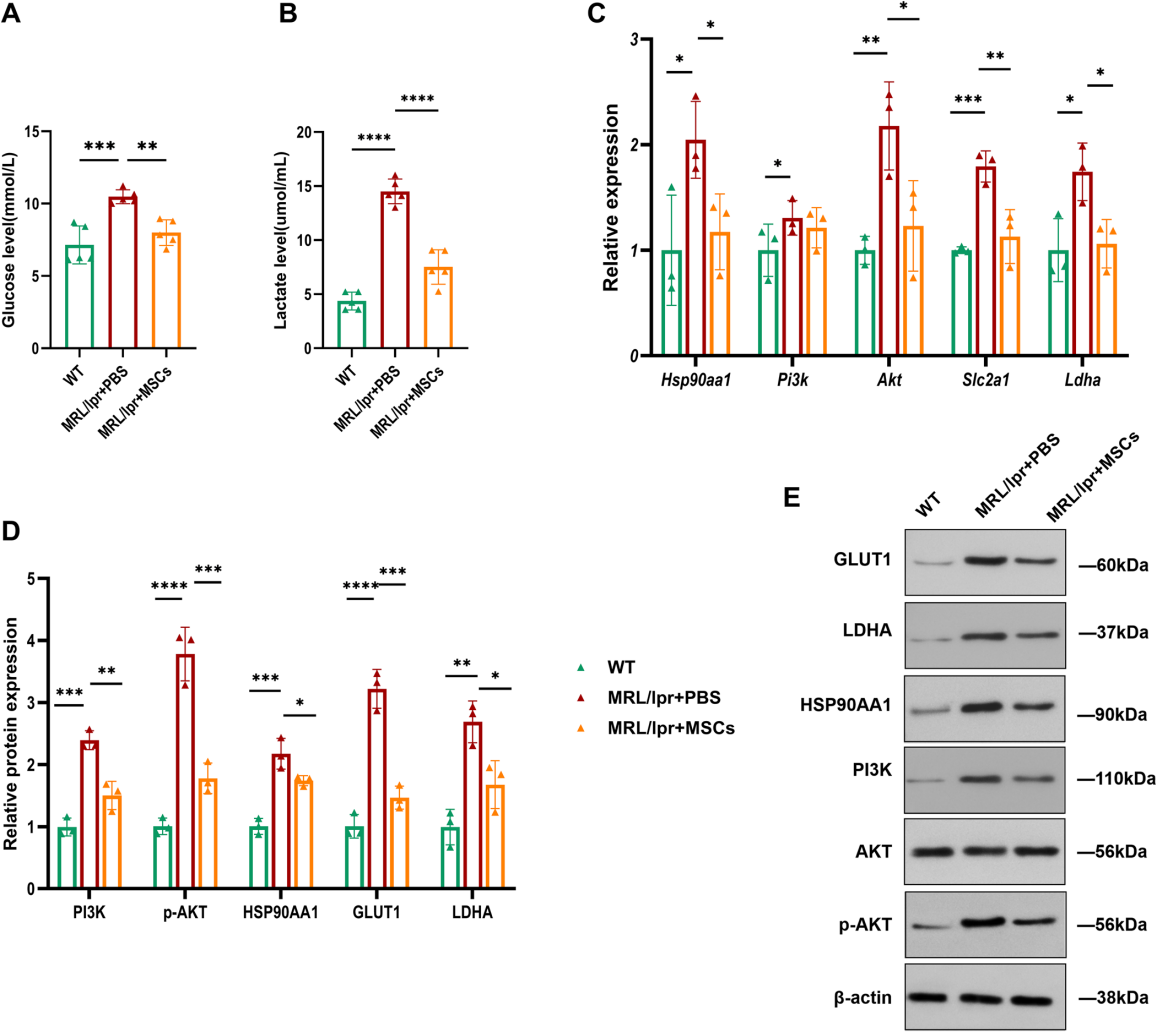
Systemic administration of hUC‐MSCs inhibited serum levels of glucose metabolites, mRNA, and protein expressions of glucose enzymes, HSP90AA1, PI3K, and AKT in the splenic CD4^+^ T cells of MRL/lpr mice. Treatment with hUC‐MSCs decreased the serum levels of (A) glucose and (B) lactate in MRL/lpr mice, *n* = 5. (C) The mRNA qualification and (D) relative protein expressions of glucose enzymes, HSP90AA1, PI3K, and AKT of splenic CD4^+^ T cells in MRL/lpr mice. (E) Representative immunoblot analysis of proteins in splenic CD4^+^ T cell in each group, *n* = 3. *p < 0.05, **p < 0.01, ***p < 0.001, ****p < 0.0001.

To elucidate the alterations in glucose metabolism and HSP90AA1/PI3K/AKT signaling pathway activity in lupus prone mice with hUC‐MSCs treatment, we analyzed the levels of glucose metabolites and relevant signaling components. ELISA analysis showed that the serum level of glucose in C57BL/6 mice group was 6.21 (6.25, 8.41) mmol/L, which increased to 10.48 ± 0.43 mmol/L in MRL/lpr mice group and declining into 7.99 ± 0.79 mmol/L with hUC‐MSCs treatment (both *p* < 0.01, Figure 10A). Lactate concentration increased from 4.37 ± 0.73 to 14.51 ± 1.02 mmol/L between normal controls and disease controls, and decreased to 7.52 ± 1.42 mmol/L in hUC‐MSCs treated group(both *p* < 0.0001, Figure 10B).

Assessing *Slc2a1* mRNA expression in splenic CD4^+^ T cells by qRT‐PCR analysis revealed a level of 1.00 ± 0.03 in C57BL/6 mice group, compared to 1.79 ± 0.12 in MRL/lpr mice group, which decreased to 1.13 ± 0.21 with hUC‐MSCs treatment (both *p* < 0.01). Quantitative qRT‐PCR measurement showed significantly increased in *Ldha* expression in MRL/lpr group compared to normal controls (1.74 ± 0.22 vs. 0.99 ± 0.24, *p* < 0.05), which reduced distinctively in lupus mice administrated with TVI of hUC‐MSCs (1.06 ± 0.18, *p* < 0.05). Sorting expression levels of relevant signaling components in HSP90AA1/PI3K/AKT pathway revealed significantly elevated levels of *Hsp90aa1*, *Pi3k*, and *Akt* in MRL/lpr mice compared to C57BL/6 mice. Notably, these levels were markedly reduced in lupus‐prone mice following treatment with hUC‐MSCs (all *p* < 0.05, Figure 10C and Table 2).

**Table 2:** Systemic administration of hUC‐MSCs exhibited a downregulated mRNA expression of *Hsp90aa1, Pi3k*, and *Akt*.

mRNA levels	WT	MRL/lpr+PBS	MRL/lpr+MSCs
	Mean ± SD *n* = 3	Mean ± SD *n* = 3	Mean ± SD *n* = 3
*Hsp90aa1*	1.00 ± 0.42	2.05 ± 0.29^#^	1.17 ± 0.42*
*Pi3k*	1.00 ± 0.20	1.31 ± 0.13^#^	1.21 ± 0.15
*Akt*	1.00 ± 0.10	2.18 ± 0.34^##^	1.23 ± 0.35*

*Note:* For comparison between WT and MRL/lpr group, ^#^ *p* < 0.05, ^##^ *p* < 0.01. For comparison between MRL/lpr + PBS and MRL/lpr + MSCs, *p < 0.05.

Western blot analysis confirmed an upregulation of GLUT1 and LDHA protein expression in splenic CD4^+^ T cells in MRL/lpr mice compared to normal controls, while a decreased in expression was observed in MRL/lpr + MSCs group (all *p* < 0.05). Quantitative WB data revealed lower detectable protein expressions of HSP90AA1, PI3K, and p‐AKT of splenic CD4^+^ T cell in normal controls, a surging expression in MRL/lpr mice, and an obviously declination after hUC‐MSCs administration (all *p* < 0.05, Figure 10D,E, Table 3).

**Table 3:** Systemic administration of hUC‐MSCs exhibited a downregulated protein expression of glucose enzymes, HSP90AA1, PI3K, and p‐AKT.

Protein expression	WT	MRL/lpr+PBS	MRL/lpr+MSCs
	Mean ± SD *n* = 3	Mean ± SD *n* = 3	Mean ± SD *n* = 3
GLUT1	1.00 ± 0.15	3.22 ± 0.25^####^	1.46 ± 0.15***
LDHA	1.00 ± 0.23	2.69 ± 0.27^##^	1.67 ± 0.31*
HSP90AA1	1.00 ± 0.11	2.17 ± 0.20^###^	1.74 ± 0.06*
PI3K	1.00 ± 0.12	2.39 ± 0.12^###^	1.50 ± 0.18**
p‐AKT	1.00 ± 0.11	3.78 ± 0.35^####^	1.78 ± 0.20***

*Note:* For comparison between WT and MRL/lpr+PBS group, ^##^ *p* < 0.01, ^###^ *p* < 0.001, ^####^ *p* < 0.0001. For comparison between MRL/lpr + PBS and MRL/lpr + MSCs, *p < 0.05, **p < 0.01,***p < 0.001.

### 3.10 Rewiring Inflammatory Microenvironment of SLE Through HSP90AA1/PI3K/AKT Signaling Pathway Downregulation in CD4^+^ T Cell During Cocultured With hUC‐MSCs

To further validate the impact of HSP90AA1/PI3K/AKT signaling pathway activity on cytokines secretion by CD4^+^ T cells cocultured with hUC‐MSCs, we conducted an vitro animal experiment to analyzed the expressions of HSP90AA1, PI3K, AKT, glucose metabolic‐related enzymes, and various subsets of CD4^+^ T cell in vitro animal experiment. Additionally, we assessed the concentrations of cytokines and glucose metabolites of supernatant of splenic CD4^+^ T cells.

**Figure 11:**
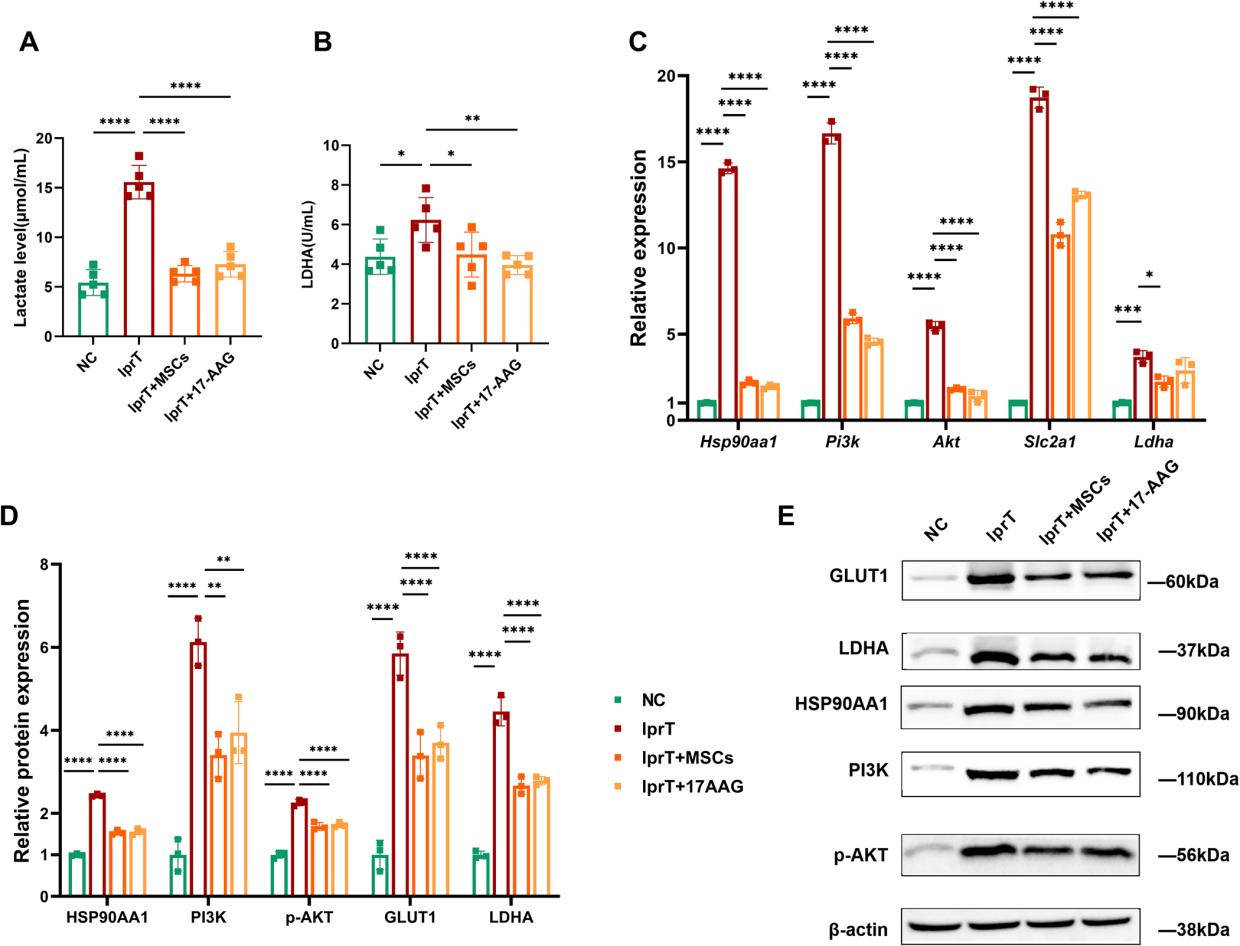
In vitro animal experiment, cocultured with hUC‐MSCs inhibited supernatant levels of glucose metabolites, mRNA, and protein expressions of glucose enzymes, HSP90AA1, PI3K, and AKT in the splenic CD4^+^ T cells of MRL/lpr mice. Cocultured with hUC‐MSCs decreased the serum levels of (A) lactate and (B) LDH in MRL/lpr mice, both *n* = 5. (C) The mRNA qualification and (D) relative protein expressions of glucose enzymes, HSP90AA1, PI3K, and AKT of splenic CD4^+^ T cells in MRL/lpr mice. (E) Representative immunoblot analysis of proteins in splenic CD4^+^ T cell in each group, all *n* = 3. *p < 0.05, **p < 0.01, ***p < 0.001, ****p < 0.0001.

The mRNA expressions and protein levels of HSP90AA1, PI3K, and AKT in CD4^+^ T cell demonstrated that hUC‐MSCs treatment downregulated HSP90AA1/PI3K/AKT signaling pathway activity (all *p* < 0.01). Additionally, analysis of glucose metabolite levels in the supernatant and the expression of glucose metabolic‐related enzymes in splenic CD4^+^ T cells revealed that HSP90AA1 inhibition led to a reduction in glucose metabolism and PI3K‐AKT pathway activity (all *p* < 0.05, Figure 11A–E, Tables 4 and 5).

**Table 4:** Inhibition of HSP90AA1 exhibited a decrease mRNA levels of glucose enzymes, *Hsp90aa1*, *Pi3k*, and *Akt* in splenic CD4^+^ T cell of lupus‐prone mice.

mRNA levels	NC	lprT	lprT+MSCs	lprT+17AAG
	Mean ± SD *n* = 3	Mean ± SD *n* = 3	Mean ± SD *n* = 3	Mean ± SD *n* = 3
*Slc2a1*	1.00 ± 0.003	18.73 ± 0.49^####^	10.79 ± 0.55****	13.06 ± 0.18^&&&&^
*Ldha*	1.00 ± 0.04	3.68 ± 0.29^###^	2.24 ± 0.27*	2.88 ± 0.60
*Hsp90aa1*	1.00 ± 0.02	14.62 ± 0.25^####^	2.18 ± 0.12****	1.94 ± 0.09^&&&&^
*Pi3k*	1.00 ± 0.01	16.64 ± 0.50^####^	5.91 ± 0.24****	4.56 ± 0.17^&&&&^
*Akt*	1.00 ± 0.01	5.46 ± 0.22^####^	1.81 ± 0.66****	1.40 ± 0.27^&&&&^

*Note:* For comparison between NC and lprT group, ^###^ *p* < 0.001, ^####^ *p* < 0.0001. For comparison between lprT and lprT+MSCs group, *p < 0.05, ****p < 0.0001. For comparison between lprT and lprT+17AAG group, ^&&&&^ *p* < 0.0001.

**Table 5:** Inhibition of HSP90AA1 exhibited a decrease protein expressions of glucose enzymes, HSP90AA1, PI3K, and p‐AKT in splenic CD4^+^ T cell of lupus‐prone mice.

Protein expression	NC	lprT	lprT+MSCs	lprT+17AAG
	Mean ± SD *n* = 3	Mean ± SD *n* = 3	Mean ± SD *n* = 3	Mean ± SD *n* = 3
GLUT1	1.00 ± 0.28	5.84 ± 0.42^####^	3.39 ± 0.45****	3.69 ± 0.32^&&&&^
LDHA	1.00 ± 0.07	4.45 ± 0.28^####^	2.79 ± 0.08****	2.67 ± 0.16^&&&&^
HSP90AA1	1.00 ± 0.02	2.42 ± 0.03^####^	1.54 ± 0.05****	1.56 ± 0.06^&&&&^
PI3K	1.00 ± 0.31	6.13 ± 0.47^####^	3.40 ± 0.44**	3.94 ± 0.61^&&^
p‐AKT	1.00 ± 0.07	2.26 ± 0.06^####^	1.69 ± 0.08****	1.72 ± 0.05^&&&&^

*Note:* For comparison between NC and lprT group, ^####^ *p* < 0.0001. For comparison between lprT and lprT + MSCs group, **p < 0.01, ****p < 0.0001. For comparison between lprT and lprT + 17AAG group, ^&&^ *p* < 0.01, ^&&&&^ *p* < 0.0001.

**Figure 12:**
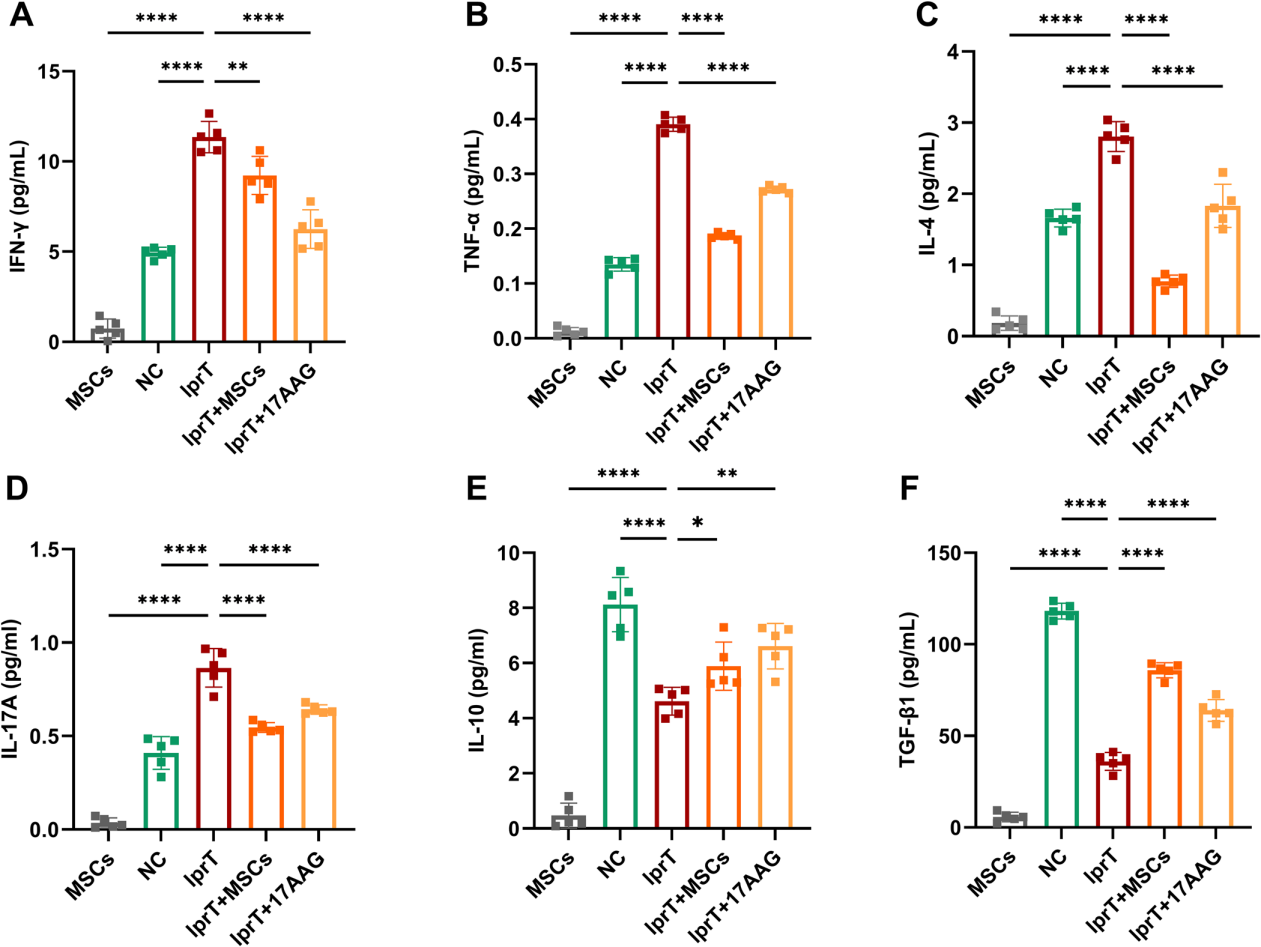
In vitro animal experiment, cocultured with hUC‐MSCs revised pro‐inflammatory profile in MRL/lpr mice. Cocultured with hUC‐MSCs inhibited the supernatant levels of (A) IFN‐γ, (B) TNF‐α, (C) IL‐4, and (D) IL‐17A, and increased the expression of (E) IL‐10 and (F) TGF‐β1. All *n* = 5. *p < 0.05, **p < 0.01, ***p < 0.001,****p < 0.0001.

The analysis of cytokines levels in supernatant from splenic CD4^+^ T cells and the examination of CD4^+^ T cell subsets have validated the complex interplay between HSP90AA1 and the inflammatory microenvironment associated with SLE. We found a significant reduction of INF‐γ (6.25 ± 0.96 vs. 11.35 ± 0.77 pg/mL), TNF‐α (0.27 ± 0.01 vs. 0.39 ± 0.01 pg/mL), IL‐4 (1.83 ± 0.27 vs. 2.81 ± 0.19 pg/mL), and IL‐17A (0.64 ± 0.01 vs. 10.92 ± 0.03 pg/mL) concentration in the supernatant of splenic CD4^+^ T cell from MRL/lpr mice pre‐cultured with 17‐AAG compared to lprT group (all *p* < 0.001, Figure 12A–D). The supernatant levels of IL‐10 (6.71 ± 0.72 vs. 8.12 ± 0.87 pg/mL) and TGF‐β1 (63.89 ± 5.27 vs. 36.09 ± 4.37 pg/mL) were significantly elevated in17AAG group (both *p* > 0.01, Figure 12E,F).

**Figure 13:**
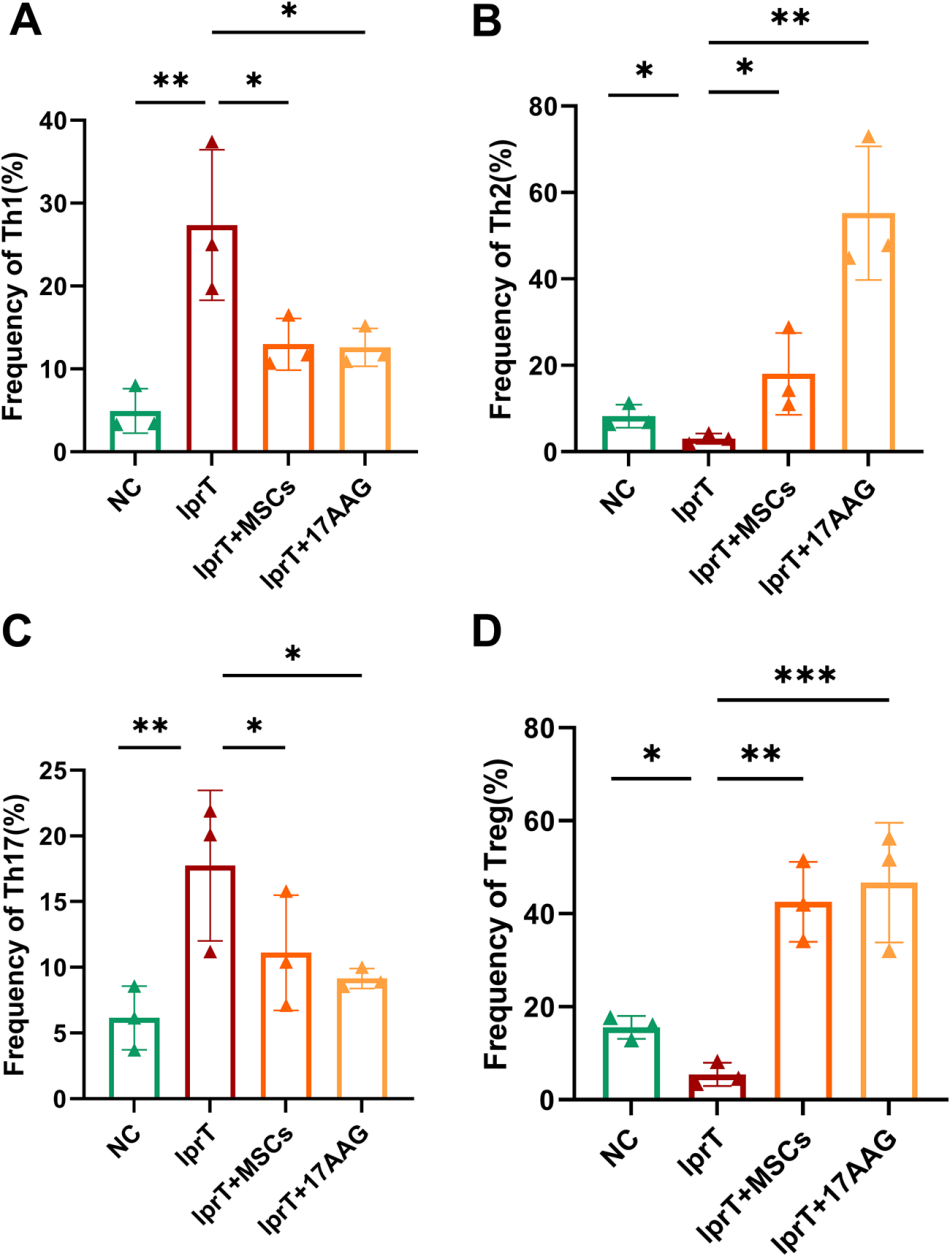
Flow cytometry analysis of splenic CD4^+^ T cell in vitro animal experiment. The frequency of (A) Th1, (B) Th2, (C) Th17, and (D) Treg cells of each mice group. All *n* = 3, *p < 0.05, **p < 0.01, ***p < 0.001.

**Figure 14:**
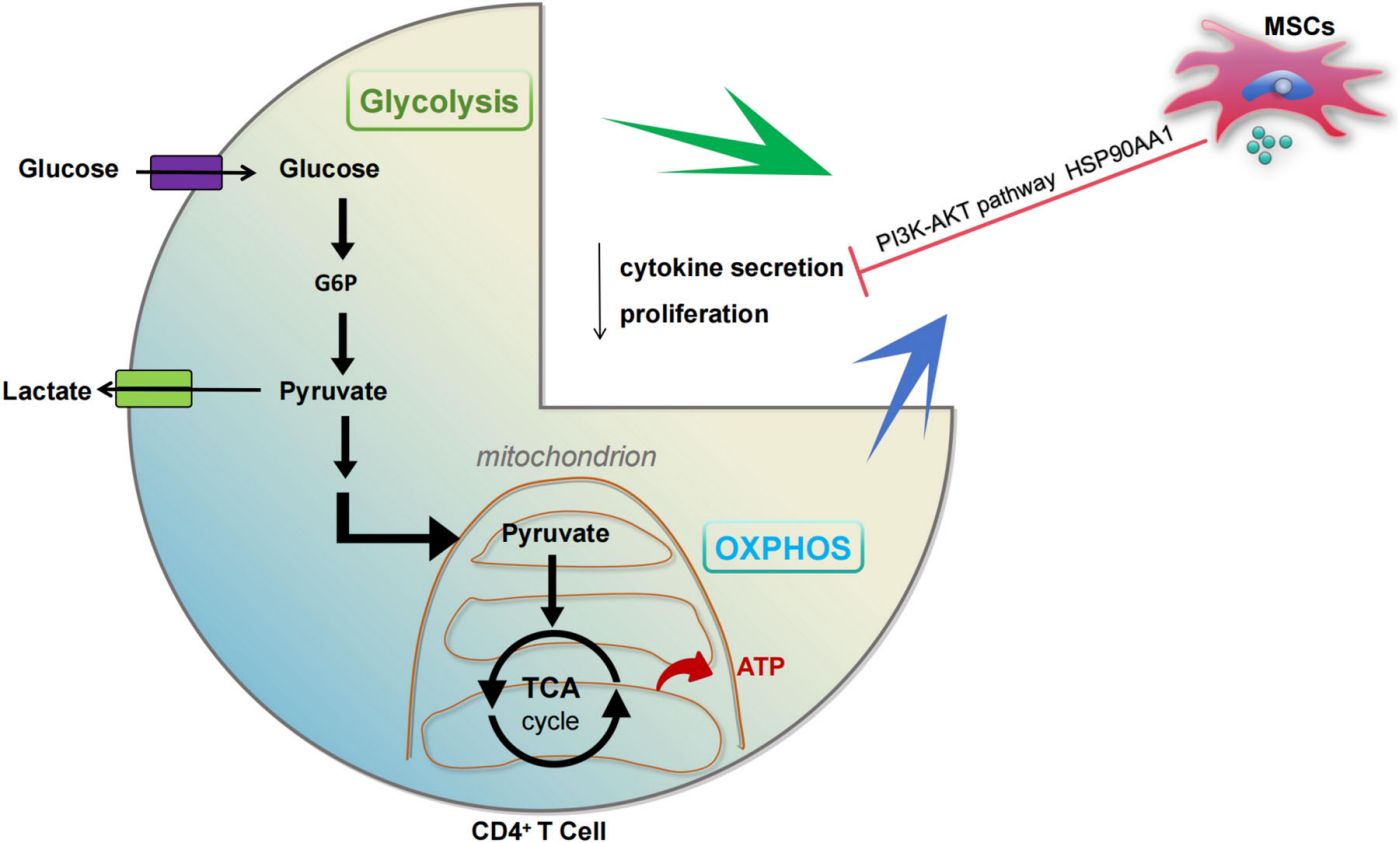
CD4^+^ T cells in SLE exhibit overactive glucose metabolism. Treatment with hUC‐MSCs effectively inhibits pro‐inflammatory milieu to promote disease remission by decreasing the expression of HSP90AA1 in the PI3K‐AKT pathway induced by CD4^+^ T cell glucose metabolic activation.

Flow cytometry analysis revealed that the frequency of Th1 and Th17 was notably higher in MRL/lpr mice compared to C57BL/6 mice, which was significantly diminished in lprT + MSCs and lprT + 17AAG groups(all *p* < 0.05, Figures 13A,C and S2). Additionally, we observed an upregulation in Th2 and Treg subpopulations in both lprT + MSCs and lprT + 17AAG groups compared to lprT group (all *p* < 0.05, Figures 13B,D, 14, and S2).

## 4 Discussion

SLE is a chronic autoimmune and inflammatory disease characterized by aberrant cytokine secretion and overactive T lymphocyte. MSCs transplantation is a promising therapy for active or refractory SLE patients owing to the strong immunomodulatory function of MSCs [26, 27, 28]. Up to now, research of MSCs transplantation therapies has largely focused on T cell proliferation, differentiation, activation, and cytokine secretion [20, 21, 29, 30]. Here, we extend these studies by analyzing changes in CD4^+^ T cell glucose metabolic capacity in lupus patients with moderate and severe activity status following hUC‐MSCs treatment. We uncovered a potential metabolic mechanism of hUC‐MSCs treatment for clinical application.

Differences in glucose metabolic capacity appear to drive the dysfunction of SLE‐CD4^+^ T cells [31, 32, 33]. Changing glucose metabolic pathways has association with the immunomodulatory activity of CD4^+^ T cells. Elevated glycolysis could support proliferation and pro‐inflammatory cytokine secretion of CD4^+^ T cells [34, 35, 36, 37]. Analyzing CD4^+^ T cell glucose metabolism, we found a significant increase in aerobic glycolysis and mitochondria oxidative consumption, as well as a pro‐inflammatory state in the supernatant in SLE compared with controls. Interestingly, the glucose metabolic rewiring was more pronounced in the moderate and severe SLE groups. Moreover, the increased glucose metabolism of CD4^+^ T cell was positively correlated with SLEDAI‐2K score and cell proliferation in lupus patients with moderate and severe activity. It is well established that the concentration of pro‐inflammatory factors parallels the severity of SLE [7, 38, 39]. Therefore, we can infer that the formation of pro‐inflammatory milieu in SLE is linked to the enhanced glucose metabolic capacity of CD4^+^ T cells.

Transplanting hUC‐MSCs has emerged as a great potential treatment for advanced or refractory lupus because of their strong immunosuppressive function, low immunogenicity, and vigorous proliferation and differentiation ability [22, 26, 40, 41, 42]. The effects of MSCs are not solely attributed to direct cell‐to‐cell interactions; rather, they also rely on the paracrine mechanisms that enhance the production of anti‐inflammatory cytokines [20, 21, 22]. However, the mechanisms by which hUC‐MSCs modulate pro‐inflammatory cytokine secretion of SLE‐CD4^+^T cell are still unknown.

Based on our previous findings and prior research [43], we conducted further analyses to investigate the effects of hUC‐MSCs treatment, including glucose metabolism assessment and cytokine detection. We found a proportional decrease in glycolytic activity and oxidative consumption of CD4^+^ T cell in the MSCT group. Moreover, we observed that hUC‐MSCs has a therapeutic function of decreasing the pro‐inflammatory cytokine secretion of CD4^+^ T cells from patients during SLE disease progression. The analysis exhibited a significantly decreased concentration of IL‐4 in the MSCT group, which is consistent with the findings of Zheng et al. [44]. In animal experiments, both the MSCs treatment group and the 17‐AAG group exhibited lower serum and supernatant levels of IL‐4 compared to lupus model mice. However, we observed an increased frequency of Th2 cells following MSCs treatment and 17‐AAG administration. The inconsistent outcome may be attributed to the extremely complex cytokine network and the limited sample size of the animals used in this study. Furthermore, consistent with findings from other studies [18, 29, 42], our research demonstrate a notable reductions of IFN‐γ, TNF‐α, and IL‐17 in the supernatant of SLE‐CD4^+^ T cells cocultured with hUC‐MSCs. IL‐10 is recognized as one of the most important inflammatory factors associated with the pathogenesis of SLE [12]. It has been established that IL‐10 is mainly produced by T cells, monocytes, B cells, macrophages, and dendritic cells [7, 12, 45]. Our result revealed the CD4^+^ T cell supernatant levels of IL‐10 decreased in SLE patients compared to HCs. This finding is inconsistent with previous research, which indicated that IL‐10 was highly expressed in the serum of patients with lupus [3]. We hypothesize that this phenomenon may be associated with the intricate regulatory network of inflammatory cells within the circulation. In our research, we assessed the expression level of IL‐10 in the culture supernatant derived from CD4^+^ T cells. In line with this observation, lower expression of IL‐10 was shown in both serum and cell supernatant in MRL/lpr mice compared to normal controls. Moreover, flow cytometry analysis indicated a significant reduction in the frequency of Th2 cells and Tregs within the MRL/lpr group compared to normal controls. Notably, Tregs are another source of IL‐10.

To further investigated the molecular mechanism of hUC‐MSCs on inflammatory microenvironment rewiring, we conducted RNA‐seq analysis. The results showed DEGs were enriched in PI3K‐AKT signaling pathway, and HSP90AA1 was the hub gene. The *HSP90AA1* gene is located on chromosome 14 and encodes the alpha isoform of heat shock protein 90αA1 (HSP90αA1), which belongs to the hsp90α subfamily [46]. HSP90 is known as an essential regulator of immune inflammatory processes associated with SLE. This is evidenced by its elevated serum expressions in lupus patients, as well as the correlation between gene polymorphisms and SLE susceptibility [47, 48, 49]. The PI3K‐AKT signaling pathway plays a key role in cell immune regulation and inflammatory response [50]. It is well established that upregulation of PI3K‐AKT signaling pathway particularly promote the progression of SLE disease [51]. Furthermore, there is increasing recognized that PI3K‐AKT signaling pathway is closely associated with glucose metabolism. The research has confirmed that activation of PI3K‐AKT signaling pathway significantly increase the expression of GLUT1, a major enzyme of glucose metabolic pathway [36, 52].

To verify the involvement of the HSP90AA1/PI3K/AKT signaling pathway as an important metabolic‐related molecular mechanism involved in hUC‐MSCs therapy aimed at improving the pro‐inflammatory microenvironment of SLE, we conducted animal experiments. The results demonstrated that hUC‐MSCs significantly decreased levels of glucose metabolites and enzymes, as well as expression of HSP90AA1, PI3K, and AKT in both vivo and vitro animal experiments. Furthermore, 17‐AAG was used as an inhibitor of HSP90AA1, which pre‐cultured with splenic CD4^+^ T cell in vitro. The findings indicated a significant downregulation of HSP90AA1, PI3K, and AKT, accompanied by inhibition of glucose metabolism and a decrease in pro‐inflammatory cytokines. Collectively, these results support the conclusion that hUC‐MSCs treatment has effects on the conversion from proinflammatory to antiinflammatory profiles in SLE, probably caused by the reduction of HSP90AA1 in PI3K‐AKT signaling pathway induced by CD4^+^ T cell glucose metabolism activation.

There are several limitations in our study. First, further research is required on the immuno‐metabolic regulation of hUC‐MSCs for mild SLE patients. Second, we added IL‐2 and IL‐7 to the CD4^+^ T cell culture medium according to culture requirements, these ILs may have influenced the results of cytokine analysis. Notably, future studies should explore healthy experimental controls involving the hUC‐MSC secretome's ability to reverse pro‐inflammatory cytokine profiles in CD4^+^ T cells, as well as different treatment modalities that inhibit glucose metabolism in SLE.

## 5 Conclusion

Our study reveals that hUC‐MSCs treatment proportionally restores the CD4^+^ T cell glucose metabolic activity in lupus patients with disease progression. For moderate and severe SLE, hUC‐MSCs effectively inhibit pro‐inflammatory milieu to promote disease remission by decreasing the expression of HSP90AA1 in the PI3K‐AKT pathway induced by CD4^+^T cell glucose metabolic activation.

## Author Contributions

**Aijing Liu:** writing – review and editing (lead), conceptualization (equal). **Lu Jin:** writing – original draft (lead), formal analysis (lead), conceptualization (equal). **Meng Ding:** investigation (lead), formal analysis (equal). **Shaoxin Cui:** data curation (lead), formal analysis (support). **Lin Yang:** visualization (lead), data curation (equal). **Jingjing He:** methodology (lead), investigation (equal), visualization (equal). **Xiaoping Wang:** data curation (equal), visualization (support). **Fei Chang:** data curation (equal), visualization (support). **Jingjing Yu:** validation (lead), methodology (equal). **Yiming Yang:** methodology (equal), validation (equal). **Hongtao Jin:** project administration (lead), writing – review and editing (equal), conceptualization (equal). **Jun Ma:** resources (lead), writing – review and editing (equal). **Min Shi:** resources (equal), writing – review and editing (equal).

## Ethics Statement

The Ethics Committee of the Second Hospital of Hebei Medical University approved this study (2021‐R392, 2023‐AE‐164).

## Conflicts of Interest

The authors declare no conflicts of interest.

## Supporting information


**Supporting Figure 1 Identification of hUC‐MSCs after co‐cultured with CD4** ^**+**^ **T cells:** (A) The expression of CD90, CD105 and CD73 were 99.94%, 99.93% and 99.94%, respectively. The expression of CD45, CD34 and HLA‐DR were 0.51%, 0.63% and 0.04%, respectively. (B) hUC‐MSCs showed spindle form such as fibroblast‐like cells in vitro culture (100 x magnification; scale bar, 200 μm).

**Supporting Figure 2 Flow cytometry analysis of splenic CD4** ^**+**^ **T cell in vitro animal experiment:** The frequency of (A)Th1, (B)Th2, (C)Th17, and (D)Treg cells of each mice group.

**Supporting Figure 3 Mitochondrial oxidative phosphorylation of CD4** ^**+**^ **T cells in lupus patients:** The (A) basal respiration, (B) maximal respiration, (C) proton leak, and (D) ATP production of CD4^+^ T cells in SLE and healthy controls. The (E) basal respiration, (F) maximal respiration, (G) proton leak, and (H) ATP production of CD4^+^ T cells in patients with different SLE activity. SLE group, n = 30; SLE‐severe group, n=7; SLE‐moderate group, n=11; SLE‐mild group, n=12; HCs, n=20. *P< 0.05, ****P< 0.0001.

**Supporting Figure 4 The capacity of aerobic glycolysis of CD4** ^**+**^ **T cells in lupus patients:** The (A) basal glycolysis and (B) basal proton efflux rate of CD4^+^ T cells in SLE and healthy controls. The (C) basal glycolysis and (D) basal proton efflux rate of CD4^+^ T cells in patients with different SLE activity. SLE group, n=30; SLE‐severe group, n=7; SLE‐moderate group, n=11; SLE‐mild group, n=12; HCs, n=20. ****P< 0.0001.

**Supporting Figure 5 Treatment with hUC‐MSCs reduced glucose metabolism of CD4** ^**+**^ **T cells from lupus:** Co‐cultured with hUC‐MSCs in different ratios decreased the (A) basal respiration, (B) maximal respiration, (C) proton leak, (D) ATP production, (E) basal glycolysis and (F) basal proton efflux rate of CD4^+^ T cells from patients with moderate and severe SLE. HCs, n=20, SLE‐MS group, n=18; T1, T2, and T3 groups, n=6, respectively. *P< 0.05, **P< 0.01, ***P< 0.001, ****P< 0.0001.

**Supporting Table 1:** The sequence of primers used in the current study.

**Supporting Table 2:** The concentration of supernatant cytokines.

## Data Availability

All raw data and code are available upon request.
