# Supplementary figures and images for: Human Umbilical Cord Mesenchymal Stem Cells Modulate Cytokine Secretion of CD4+ T Cell in Systemic Lupus Erythematosus by Inhibiting HSP90AA1 in the Glucose‐Activated PI3K‐AKT Pathway
Source: Immun Inflamm Dis. 2025 Aug 13;13(8):e70239. doi: 10.1002/iid3.70239 (PMC12344575; doi:10.1002/iid3.70239)

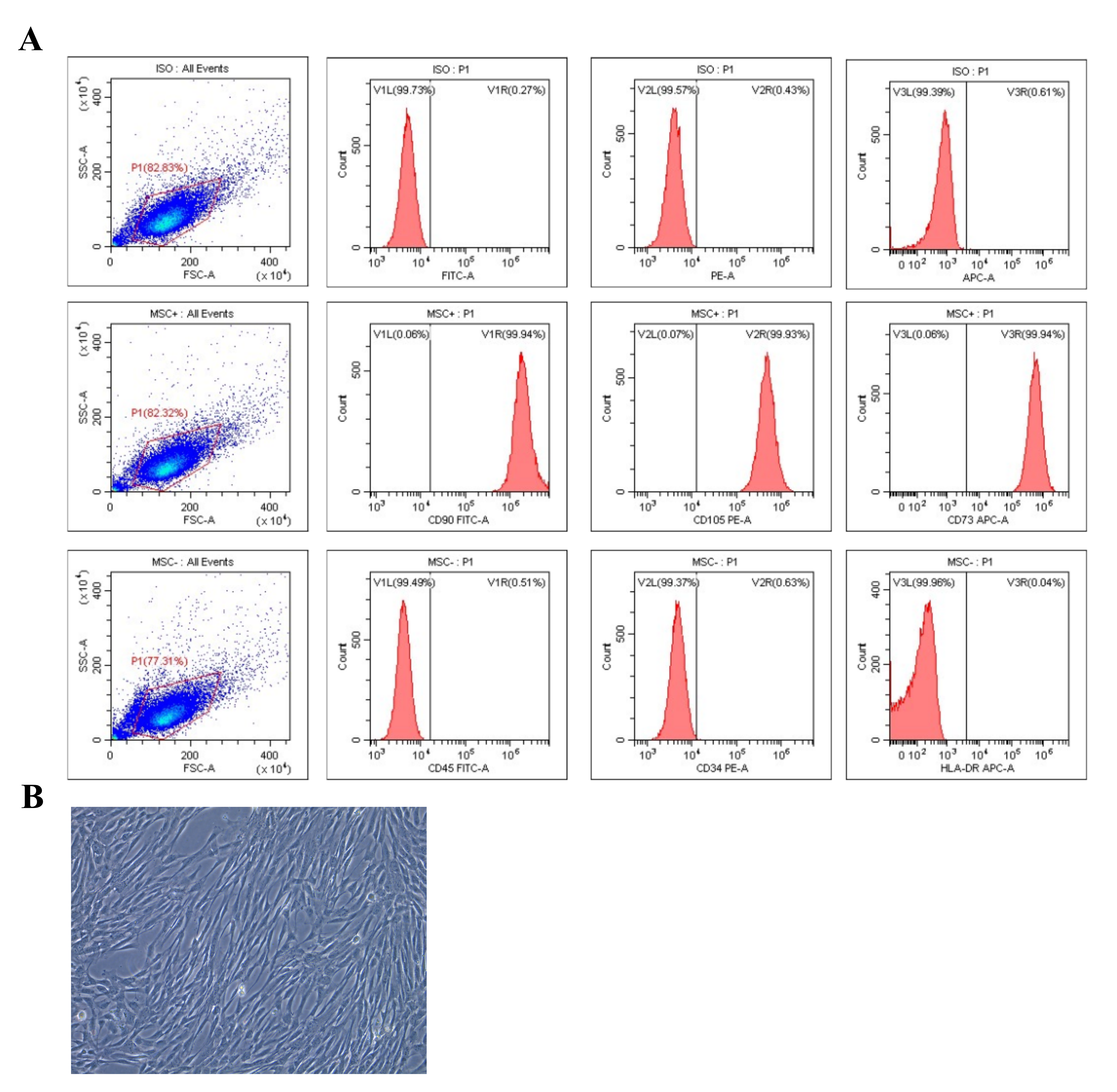

Supplement: Supplementary file 1 — Supporting Figure 1 Identification of hUC‐MSCs after co‐cultured with CD4 + T cells: (A) The expression of CD90, CD105 and CD73 were 99.94%, 99.93% and 99.94%, respectively. The expression of CD45, CD34 and HLA‐DR were 0.51%, 0.63% and 0.04%, respectively. (B) hUC‐MSCs showed spindle form such as fibroblast‐like cells in vitro culture (100 x magnification; scale bar, 200 μm). [file IID3-13-e70239-s007.tif]

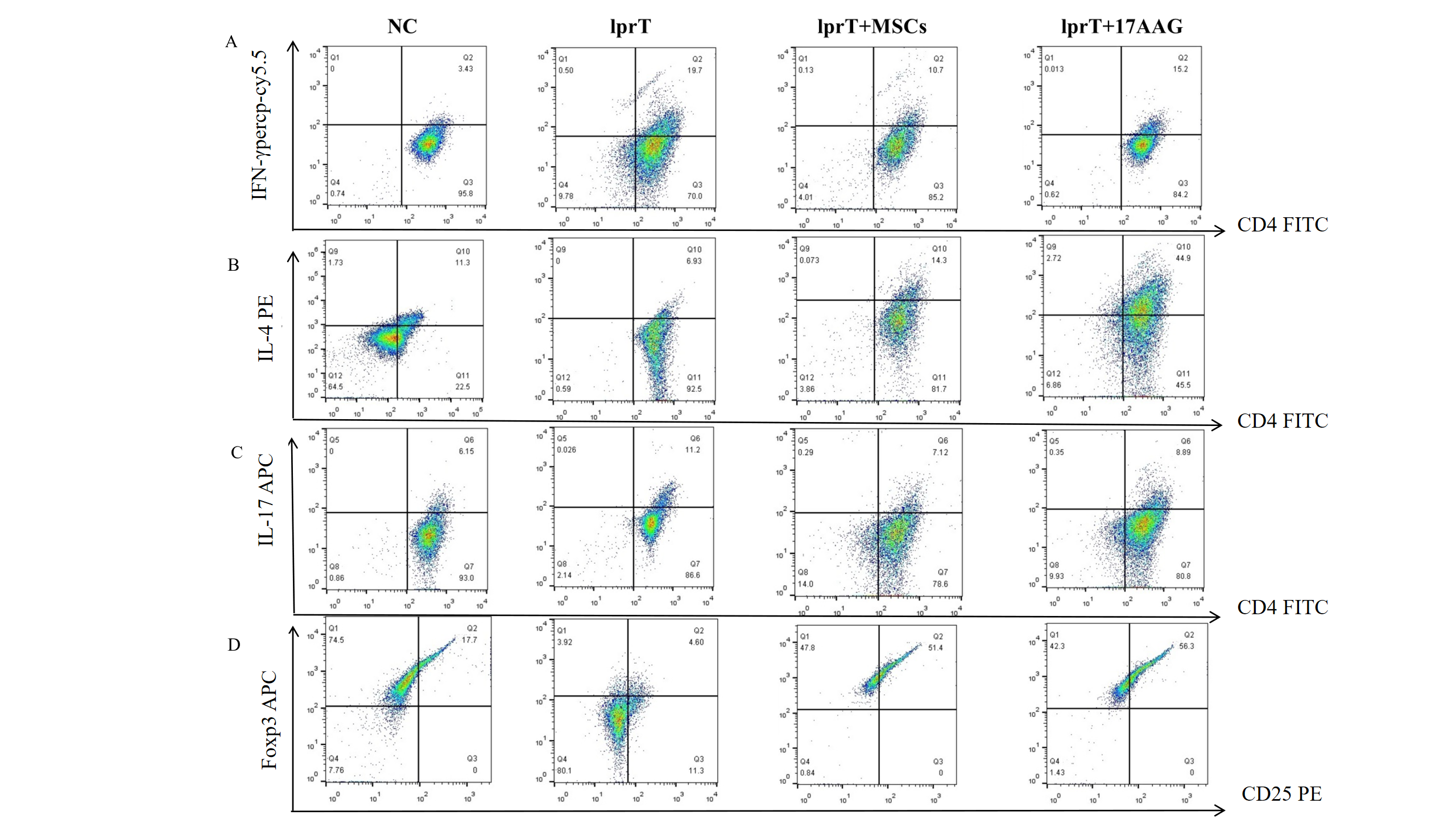

Supplement: Supplementary file 2 — Supporting Figure 2 Flow cytometry analysis of splenic CD4 + T cell in vitro animal experiment: The frequency of (A)Th1, (B)Th2, (C)Th17, and (D)Treg cells of each mice group. [file IID3-13-e70239-s004.tif]

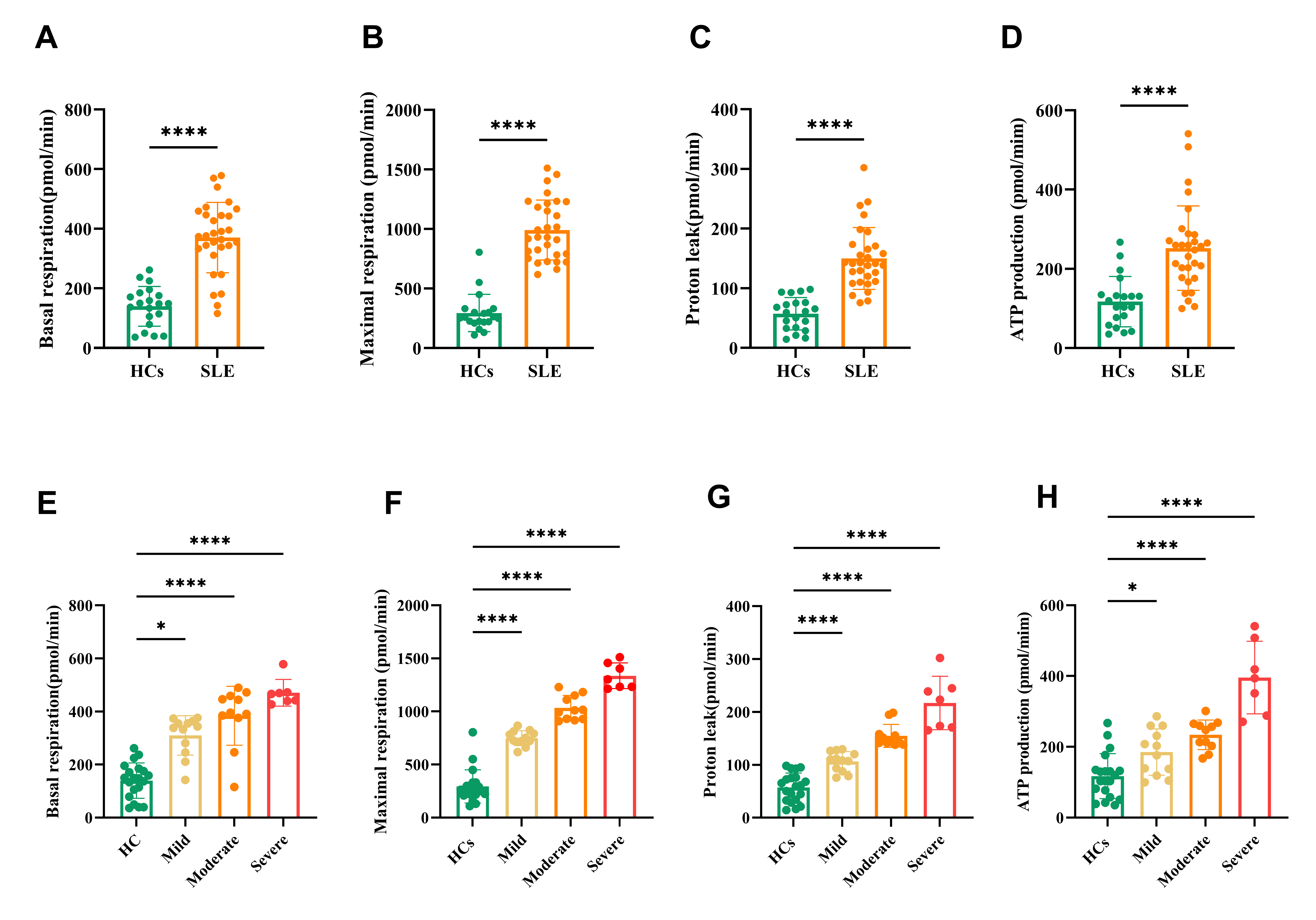

Supplement: Supplementary file 3 — Supporting Figure 3 Mitochondrial oxidative phosphorylation of CD4 + T cells in lupus patients: The (A) basal respiration, (B) maximal respiration, (C) proton leak, and (D) ATP production of CD4+ T cells in SLE and healthy controls. The (E) basal respiration, (F) maximal respiration, (G) proton leak, and (H) ATP production of CD4+ T cells in patients with different SLE activity. SLE group, n = 30; SLE‐severe group, n=7; SLE‐moderate group, n=11; SLE‐mild group, n=12; HCs, n=20. *P< 0.05, ****P< 0.0001. [file IID3-13-e70239-s005.tif]

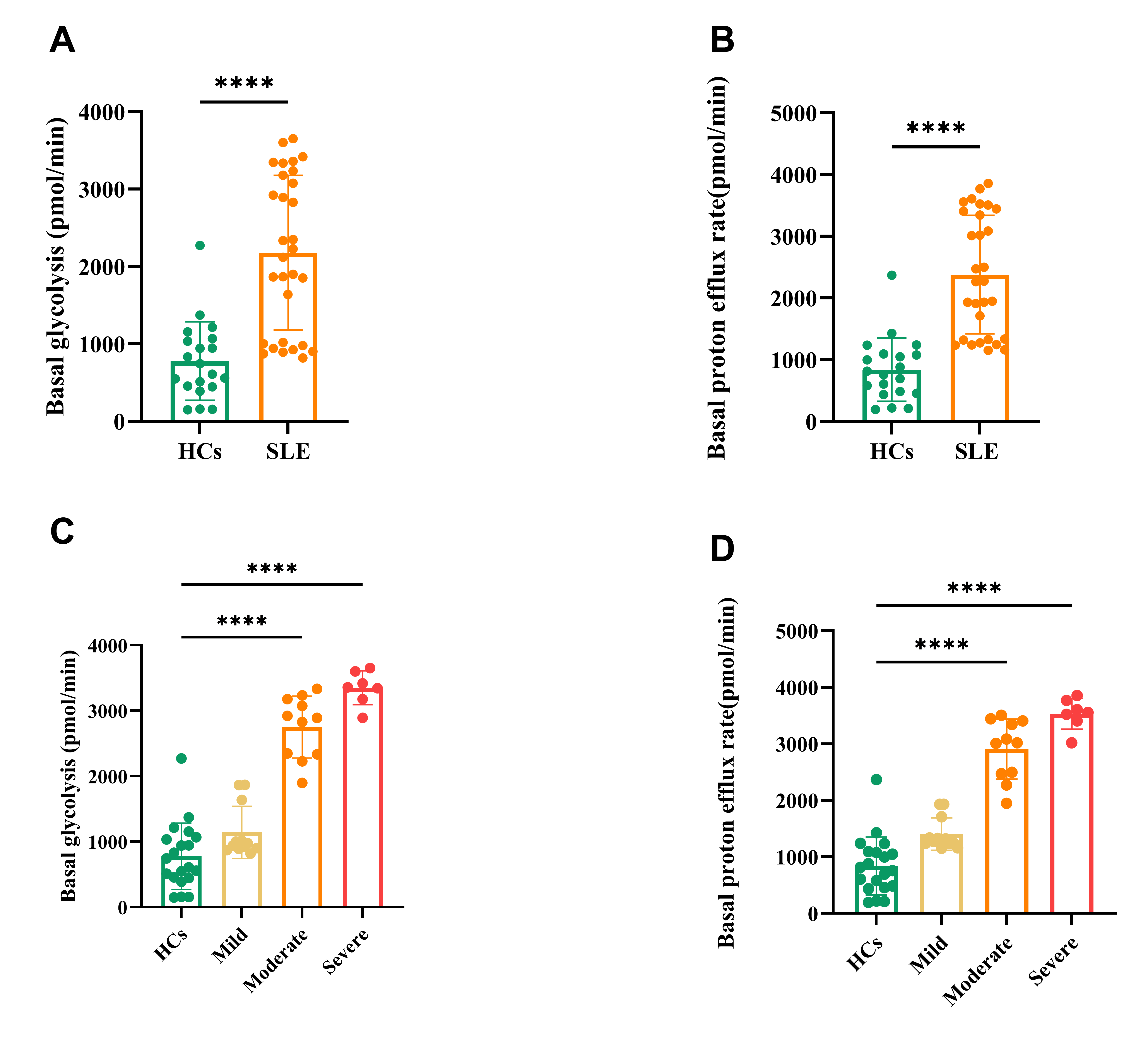

Supplement: Supplementary file 4 — Supporting Figure 4 The capacity of aerobic glycolysis of CD4 + T cells in lupus patients: The (A) basal glycolysis and (B) basal proton efflux rate of CD4+ T cells in SLE and healthy controls. The (C) basal glycolysis and (D) basal proton efflux rate of CD4+ T cells in patients with different SLE activity. SLE group, n=30; SLE‐severe group, n=7; SLE‐moderate group, n=11; SLE‐mild group, n=12; HCs, n=20. ****P< 0.0001. [file IID3-13-e70239-s003.tif]

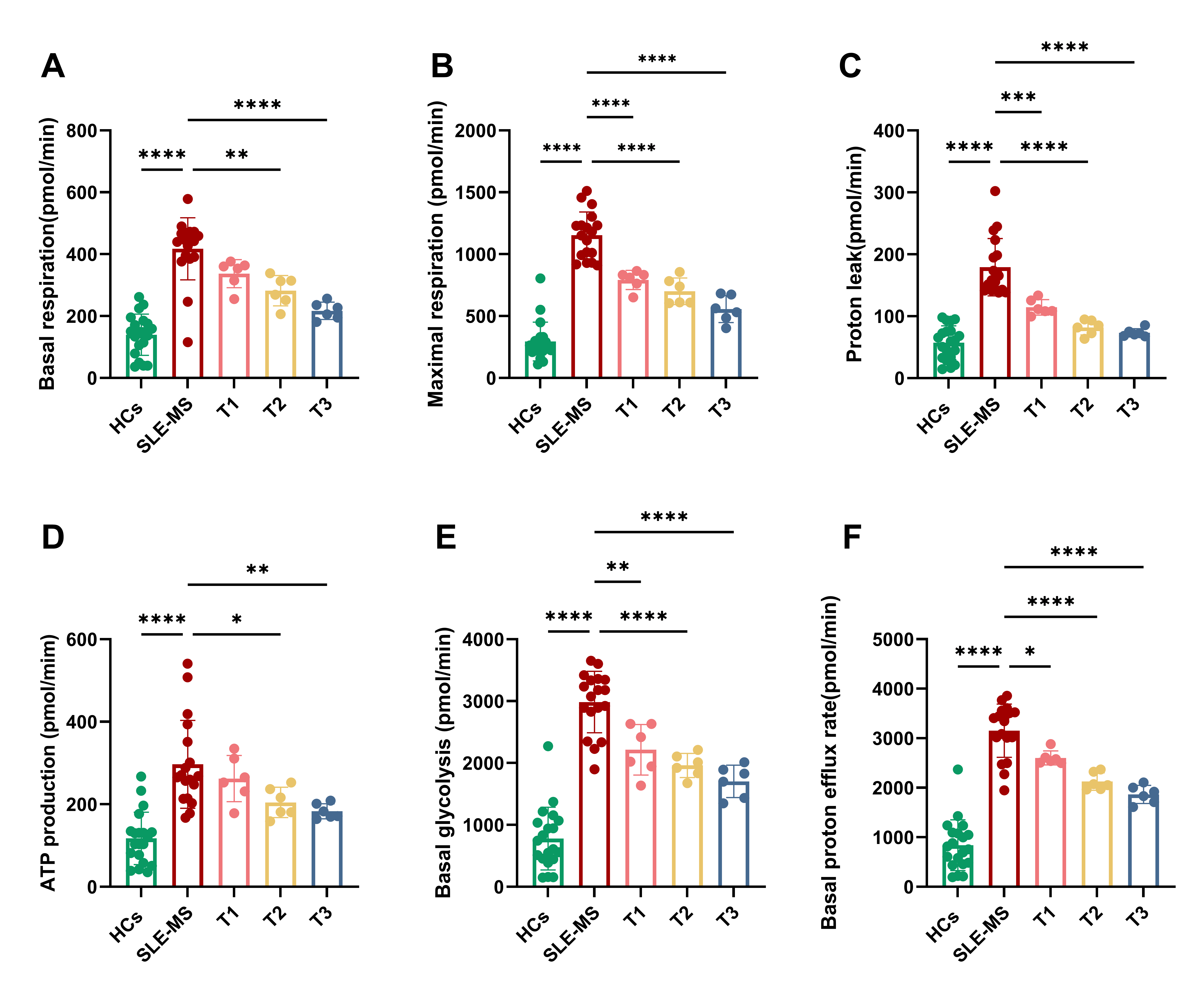

Supplement: Supplementary file 5 — Supporting Figure 5 Treatment with hUC‐MSCs reduced glucose metabolism of CD4 + T cells from lupus: Co‐cultured with hUC‐MSCs in different ratios decreased the (A) basal respiration, (B) maximal respiration, (C) proton leak, (D) ATP production, (E) basal glycolysis and (F) basal proton efflux rate of CD4+ T cells from patients with moderate and severe SLE. HCs, n=20, SLE‐MS group, n=18; T1, T2, and T3 groups, n=6, respectively. *P< 0.05, **P< 0.01, ***P< 0.001, ****P< 0.0001. [file IID3-13-e70239-s001.tif]
