# Supplementary material for: Human Umbilical Cord Mesenchymal Stem Cells Modulate Cytokine Secretion of CD4+ T Cell in Systemic Lupus Erythematosus by Inhibiting HSP90AA1 in the Glucose‐Activated PI3K‐AKT Pathway
Source: Immun Inflamm Dis. 2025 Aug 13;13(8):e70239. doi: 10.1002/iid3.70239 (PMC12344575; doi:10.1002/iid3.70239)
Supplement: Supplementary file 6 — Supporting Table 1: The sequence of primers used in the current study. [file IID3-13-e70239-s002.docx]

**SUPPLEMENTARY TABLE 1** The sequence of primers used in the current study.

| Primer name | Sequence (5′→3′) |
| --- | --- |
| *HSP90AA1* | Forward:5’-GAAGGAATTTGAGGGGAAGACTTTA-3  Reverse:5’-TGCCATGTAACCCATTGTTGAG-3 |
| Mus *Hsp90aa1* | Forward:5’-TCAGAGCTGTTGCGGTACTA-3  Reverse:5’-GCTTTCGGAGACGTTCCACA-3 |
| Mus *Ldha* | Forward:5’-CTTCTCCTCGCCAGTCGC-3  Reverse:5’-CAACAGCACCAACCCCAA-3 |
| Mus *Slc2a1* | Forward:5’-CATCCACCACACTCACCACG-3  Reverse:5’-GAGAAGCCCATAAGCACAGCA-3 |
| Mus *Pi3k* | Forward:5’-AATGCACGGCGATTACACTC-3  Reverse:5’-GGACACTGGGTAGAGCAACT-3 |
| Mus *Akt* | Forward:5’-CTGCCCTTCTACAACCAGGA-3  Reverse:5’-CATACACATCCTGCCACACG-3 |
| β-actin | Forward: 5’-GGCTGTATTCCCCTCCATCG-3′  Reverse: 5’-CCAGTTGGTAACAATGCCATGT-3 |
