# Supplementary material for: Human Umbilical Cord Mesenchymal Stem Cells Modulate Cytokine Secretion of CD4+ T Cell in Systemic Lupus Erythematosus by Inhibiting HSP90AA1 in the Glucose‐Activated PI3K‐AKT Pathway
Source: Immun Inflamm Dis. 2025 Aug 13;13(8):e70239. doi: 10.1002/iid3.70239 (PMC12344575; doi:10.1002/iid3.70239)
Supplement: Supplementary file 7 — Supporting Table 2: The concentration of supernatant cytokines. [file IID3-13-e70239-s006.docx]

**SUPPLEMENTARY TABLE 2** The concentration of supernatant cytokines.

| Cytokine  (pg/ml) | HCs  N=6 | SLE-MS  N=6 | MSCT (1:10)  N=6 |
| --- | --- | --- | --- |
| IL-2R α | 653.160±36.132 | 1977.000±94.820^A^ | 1329.667±47.322^B^ |
| MIG | 2855.567±1213.654 | 4013.667±595.435 | 2925.333±93.923^B^ |
| IFN-α2 | 66.247±12.768 | 76.977±5.558 | 76.697±2.311 |
| IFN-γ | 3306.000±782.908 | 7376±587.882^A^ | 3698.267±155.149^B^ |
| SDF-1α | 257.920±9.601 | 272.870±11.667^a^ | 266.087±6.744 |
| IL-1Rα | 6669.110±6514.377 | 5777.667±846.063 | 11723.667±674.357^B^ |
| MCP-3 | 14.683±0.826 | 2518.333±124.512^A^ | 1854.857±70.663^B^ |
| IL-16 | 125.763±1.191 | 198.470±10.464^A^ | 145.947±4.324^B^ |
| IL-12p40 | 551.060±27.333 | 880.667±39.866^A^ | 759.240±25.937^B^ |
| TNF-β | 3755.667±168.419 | 7180.000±143.376^A^ | 5067.000±498.954^B^ |
| IL-5 | 891.427±162.319 | 1173.003±12.432^A^ | 1055.333±10.894^B^ |
| GM-CSF | 2395.333±289.178 | 5271.000±94.565^A^ | 5075.000±113.677^B^ |
| MIF | 2390.333±282.085 | 4859.667±655.834^A^ | 2657.333±183.183^B^ |
| TNF-α | 19708.000±1496.285 | 31407.000±2255.222^A^ | 24579.333±2118.987^B^ |
| RANTES | 3617.000±51.439 | 5962.667±258.606^A^ | 4097.667±77.488^B^ |
| IL-2 | 14927.000±2180.444 | 14537.667±915.878 | 14195.333±1728.347 |
| IL-1β | 37.873±5.733 | 673.877±102.825^A^ | 383.227±35.284^B^ |
| IL-18 | 60.113±4.694 | 98.507±8.538^A^ | 75.270±2.871^B^ |
| Basic FGF | 161.213±4.909 | 259.690±18.129^A^ | 212.733±1.972^B^ |
| VEGF | 396.227±12.306 | 511.720±18.751^A^ | 414.253±15.856^B^ |
| β-NGF | 64.760±5.117 | 55.553±4.764^A^ | 52.883±5.688 |
| PDGF-BB | 421.977±10.573 | 592.420±24.646^A^ | 552.753±11.959^B^ |
| IP-10 | 1185.763±296.276 | 4501.000±1901.094^A^ | 3228.000±255.685 |
| IL-13 | 569.403±177.676 | 1420.933±216.079^A^ | 1124.667±110.514^b^ |
| IL-4 | 24.403±1.041 | 34.740±1.577^A^ | 32.063±1.311^b^ |
| MCP-1 | 228.120±8.359 | 2346.000±224.121^A^ | 2043.000±73.097^B^ |
| IL-8 | 6934.333±463.337 | 19633.000±1532.641^A^ | 18289.333±411.914 |
| IL-10 | 1213.333±50.119 | 730.333±54.689^A^ | 986.463±191.312^b^ |
| G-CSF | 12691.000±1025.274 | 22967.667±2338.526^A^ | 100719.000±16968.744^B^ |
| GRO-α | 2400.000±127.056 | 19313.333±3682.406^A^ | 17811.667±1041.704 |
| HGF | 596.503±29.769 | 907.423±96.160^A^ | 1160.480±107.523^B^ |
| IL-1α | 525.487±42.203 | 829.073±111.972^A^ | 767.967±35.796 |
| IL-3 | 185.080±5.520 | 498.413±61.428^A^ | 277.293±8.807^B^ |
| SCF | 178.077±17.918 | 292.403±39.416^A^ | 267.653±11.342 |
| TRAIL | 115.000±9.273 | 623.827±33.222^A^ | 430.827±49.050^B^ |
| M-CSF | 73.360±11.004 | 137.283±5.543^A^ | 93.230±0.404^B^ |
| CTACK | 158.220±12.154 | 642.320±16.066^A^ | 718.303±1.715^B^ |
| IL-15 | 716.793±5.847 | 776.977±41.699^A^ | 744.820±32.858 |
| IL-7 | 1234.623±862.081 | 3390.000±848.794^A^ | 1683.667±441.247^B^ |
| IL-12p70 | 19.870±3.543 | 25.700±2.473^A^ | 23.040±3.030 |
| IL-17 | 6783.333±2805.160 | 13554.333±1817.025^A^ | 10983.000±741.559^b^ |
| IL-9 | 1570.333±290.027 | 1947.000±134.022^a^ | 1607.493±116.536^B^ |
| SCGF-β | 12902.000±2336.887 | 7348.000±682.668^A^ | 8604.667±1214.399^b^ |

Note: a, compared with healthy controls, *p*<0.05; A, compared with healthy controls *p* < 0.01; b, compared with SLE group, *p*<0.05; B, compared with SLE group, *p*<0.01.
